# Effects of different anti-osteoporosis medications on re-fracture risk, bone mineral density, and functional recovery following hip fragility fracture surgery: a systematic review and network meta-analysis

**DOI:** 10.3389/fendo.2026.1949781

**Published:** 2026-10-20

**Authors:** Jingnan Tian, Hang Zhang, Yuchi Lu, Hailong Wu, Yan Lu, Dongxu Yu, Lianhe Yu, Zongjian Luo

**Affiliations:** 1 College of Chinese Medicine, Changchun University of Chinese Medicine, Changchun, China; 2 Department of Orthopedics, Affiliated Hospital of Changchun University of Chinese Medicine, Changchun, China; 3 Department of Orthopedics, Third Affiliated Hospital of Changchun University of Chinese Medicine, Changchun, China

**Keywords:** anti-osteoporosis medications, fragility hip fracture, hip bone mineral density, network meta-analysis, refracture

## Abstract

**Background:**

Patients undergoing surgery for fragility hip fracture remain at high risk of refracture and poor functional recovery. This study compared the effects of commonly used anti-osteoporosis medications on postoperative outcomes.

**Methods:**

PubMed, Embase, Web of Science, and the Cochrane Library were searched from January 1, 2015, to April 15, 2026. Randomized and non-randomized comparative studies were eligible. Bayesian random-effects consistency-model network meta-analyses assessed refracture, hip bone mineral density expressed as g/cm^2^ or T-scores, Harris Hip Score, and visual analog scale scores. Leave-one-out sensitivity analyses were conducted for all outcomes. For HHS and VAS, RCT-only analyses were performed when the available RCTs formed connected networks, and analyses restricted to exactly 12 months of follow-up were conducted where feasible. The certainty of evidence was assessed using the GRADE approach.

**Results:**

Eighteen studies involving 2,414 patients were included. Comparative effects varied by outcome and follow-up duration, with no consistent evidence of treatment superiority. Ranking patterns suggested relatively favorable signals for ZOL and DEN in 12-month hip refracture, ZOL and ALN in 12-month overall refracture, and TPTD and DEN in 24-month overall refracture, but were interpreted as descriptive summaries. BMD ranking patterns varied according to reporting format, and no clear differences were found for BMD, HHS, or VAS. Leave-one-out sensitivity analyses showed broadly stable findings, and the additional RCT-only and exact 12-month analyses for HHS and VAS were generally consistent with the primary analyses. GRADE assessments indicated limited certainty of evidence across outcomes.

**Conclusion:**

Anti-osteoporosis medications showed outcome- and time-specific efficacy patterns, but no consistent superiority was demonstrated. ZOL, DEN, and ALN showed relatively favorable signals for some early refracture outcomes, whereas TPTD showed a more favorable pattern for longer-term bone mass restoration. Given the limited and predominantly low-certainty evidence, these findings should be interpreted as contextual efficacy signals rather than treatment recommendations.

**Systematic review registration:**

https://www.crd.york.ac.uk/PROSPERO/view/CRD420261363237, identifier CRD420261363237.

## Introduction

Fragility hip fracture is one of the most severe fracture types in older patients with osteoporosis. It is usually caused by low-energy trauma and is closely associated with increased mortality, limited functional recovery, reduced quality of life, and greater long-term care burden (1, 2). According to Global Burden of Disease 2019 estimates, the global incidence and prevalence rates of hip fracture among adults aged ≥55 years were 681.35 and 1,191.39 per 100,000 population, respectively; both rates increased markedly with age and were higher among women (3). International epidemiological data further suggest that the number of hip fractures may nearly double by 2050, highlighting the growing medical and social care burden (4). Surgical treatment can restore fracture stability, promote early mobilization, and reduce complications related to prolonged bed rest; however, surgery itself does not correct the underlying pathological state of osteoporosis. For patients after surgery for a fragility hip fracture, the risk of refracture, insufficient improvement in hip bone mass, persistent pain, and poor recovery of hip joint function remain important factors affecting long-term prognosis. Therefore, secondary prevention through postoperative osteoporosis management is important for improving long-term outcomes.

Anti-osteoporosis treatment is an important component of secondary prevention after surgery for fragility hip fracture (5, 6). Commonly used agents include antiresorptive therapies, such as bisphosphonates (zoledronic acid, alendronate, and risedronate) and denosumab, as well as the anabolic agent teriparatide. Bisphosphonates and denosumab mainly suppress osteoclast-mediated bone resorption, whereas teriparatide promotes bone formation; these complementary actions may improve bone mineral density and reduce fracture risk (7–9). Previous studies have shown that zoledronic acid after hip fracture can reduce the risk of new clinical fractures and may confer a survival benefit (10–12). Teriparatide has also been investigated for postoperative outcomes after hip and other osteoporotic fractures; however, existing systematic reviews have not reached consistent conclusions regarding whether it clearly improves fracture healing, pain relief, or functional recovery (13–15). Therefore, in the specific clinical context after surgery for a fragility hip fracture, the relative efficacy and clinical applicability of different anti-osteoporosis medications require further comparison.

Many systematic reviews and network meta-analyses have investigated anti-osteoporosis medications in patients with osteoporosis or osteoporotic fractures, but the existing evidence provides limited direct guidance for patients after surgery for a fragility hip fracture (13–21). First, previous multi-drug comparative studies have largely focused on postmenopausal women with osteoporosis and have mainly examined osteoporosis-related outcomes such as fracture risk reduction, safety, and improvement in bone mineral density at different skeletal sites, with relatively less attention to rehabilitation-related outcomes such as hip joint functional recovery and pain control after hip fracture surgery (16–18). Some studies focused on bisphosphonates, whereas others included several anti-osteoporosis medications but mainly addressed bone mineral density improvement and fracture prevention in postmenopausal women with osteoporosis (19, 20). Other studies compared only selected regimens, such as denosumab, raloxifene, romosozumab, and teriparatide, rather than specifically targeting patients after surgery for fragility hip fracture (21). Second, although some systematic reviews have focused on the role of teriparatide alone in the treatment of hip, pelvic, and other osteoporotic fractures, their interventions were relatively concentrated and their outcomes mainly involved fracture healing indicators, treatment failure, pain scores, and functional outcomes; therefore, they cannot fully compare the relative efficacy of multiple clinically used anti-osteoporosis medications in patients after surgery for fragility hip fracture (13–15). Meanwhile, functional status, pain control, quality of life, and care needs after hip fracture surgery are closely related to patient recovery and long-term prognosis (22, 23). Thus, although existing evidence supports the importance of anti-osteoporosis treatment, direct and systematic comparative evidence remains lacking regarding how to select medication regimens for patients after surgery for fragility hip fracture under different clinical goals.

Network meta-analysis can integrate both direct and indirect evidence, compare the relative efficacy of multiple interventions when sufficient head-to-head studies are lacking, and generate probability rankings for different treatment regimens (24). Therefore, this systematic review and Bayesian network meta-analysis aimed to compare the relative effects of commonly used anti-osteoporosis medications after surgery for fragility hip fracture on postoperative refracture, hip bone mineral density, hip function, and pain. The goal was to provide clinically relevant comparative evidence for treatment selection in postoperative orthopaedic management and secondary fracture prevention.

## Methods

### Literature search strategy

This study was conducted and reported using systematic review and network meta-analysis methods in accordance with the Preferred Reporting Items for Systematic Reviews and Meta-Analyses extension for Network Meta-Analyses (PRISMA-NMA) reporting guideline (25). It was registered in the International Prospective Register of Systematic Reviews (PROSPERO; registration number: CRD420261363237). Databases were searched from January 1, 2015, to April 15, 2026. The 2015 cutoff was pragmatically selected to capture approximately the most recent decade, during which relevant surgical and postoperative approaches were considered broadly stable and comparable. The cutoff was not intended to represent a discrete change in surgical practice, treatment standards, or methodology in 2015, nor to suggest that earlier studies were invalid or clinically irrelevant. Earlier studies were excluded solely because they fell outside the defined search period.The databases searched included PubMed, Embase, Web of Science, and the Cochrane Library. Search terms mainly included “hip fracture”, “femoral neck fracture”, “intertrochanteric fracture”, “osteoporosis”, “anti-osteoporosis treatment”, “zoledronic acid”, “denosumab”, “teriparatide”, “alendronate”, “risedronate”, “refracture”, “hip bone mineral density”, “bone mineral density”, “Harris hip score”, and “visual analog scale”. Medical subject headings and free-text terms were combined, and search strategies were adjusted according to the characteristics of each database. The references of included studies and relevant reviews were also screened to identify potentially missed studies. The detailed search strategy is provided in **Supplementary Table 1**.

### Inclusion and exclusion criteria

The inclusion criteria were as follows: (1) the study population comprised patients with fragility hip fracture who underwent surgical treatment; (2) the interventions included zoledronic acid (ZOL), denosumab (DEN), teriparatide (TPTD), alendronate (ALN), risedronate (RIS), pre-fracture alendronate exposure followed by postoperative teriparatide treatment (ALN+TPTD), or background therapy control (CTRL), defined as calcium and/or vitamin D treatment, with or without placebo; (3) the study included at least two or more comparison groups and allowed extraction of outcome data by group; (4) at least one outcome of interest was reported, including refracture rate, hip bone mineral density (BMD), Harris Hip Score (HHS), or visual analog scale (VAS) score. Refracture outcomes included 12-month hip refracture rate, 12-month overall refracture rate, and 24-month overall refracture rate. For this review, BMD was restricted to measurements at the hip. Areal BMD reported in g/cm^2^ and BMD-derived T-scores were treated as distinct outcomes and analyzed separately; (5) the follow-up duration for outcome assessment was at least 6 months, and outcome data at the corresponding follow-up time point were extractable; (6) the study design was a randomized controlled trial or a non-randomized comparative study. Non-randomized comparative studies included prospective cohort studies, retrospective cohort studies, retrospective observational studies, and other retrospective studies with clearly defined comparison groups; and (7) only articles published in English were included.

The exclusion criteria were as follows: (1) animal experiments, basic research, case reports, reviews, conference abstracts, study protocols, commentaries, or guidelines; (2) studies in which the population was not patients after surgery for fragility hip fracture, or in which data for patients with fragility hip fracture could not be extracted separately; (3) studies in which the intervention or control did not meet the prespecified criteria, or in which specific anti-osteoporosis drug categories could not be clearly distinguished; (4) studies that did not report outcomes of interest, or in which outcome data were incomplete and could not be converted; (5) studies with unclear follow-up duration or for which the follow-up time point corresponding to the outcome data could not be determined; and (6) duplicate publications or studies with clearly overlapping data sources. For duplicate publications, priority was given to the study with the larger sample size, more complete follow-up, or more fully reported data.

### Study selection and data extraction

Two researchers independently conducted study selection and data extraction. Titles and abstracts were first screened to exclude studies that clearly did not meet the inclusion criteria. Full texts were then reviewed, and the final included studies were determined according to the inclusion and exclusion criteria. Disagreements between the two researchers were resolved through discussion or, when necessary, by consultation with a third researcher.

Extracted information included the first author, publication year, country or region, study design, sample size, fracture type, baseline patient characteristics, baseline BMD or osteoporosis severity, surgical procedure, intervention, comparator, treatment-initiation timing, follow-up duration, calcium/vitamin D supplementation, outcome measures, and, where applicable, the measurement scale, anatomical measurement site and laterality, measurement method or device, and outcome assessment time point. These variables were prespecified as potential effect modifiers relevant to the assessment of transitivity. For dichotomous outcomes, the number of events and the total number of participants in each group were extracted. For continuous outcomes, the sample size, mean, and standard deviation for each group were extracted. BMD reported in g/cm^2^ and T-scores were analyzed as separate continuous outcomes using mean differences within each scale. When the standard deviation was not directly reported, it was converted from the standard error, confidence interval, P value, or other available statistics.

For refracture outcomes, the number of refracture events and the total number of patients in each intervention group at the corresponding follow-up time point were extracted. According to the reported refracture site and follow-up duration, refracture outcomes were categorized into 12-month hip refracture rate, 12-month overall refracture rate, and 24-month overall refracture rate and analyzed separately. Hip refracture rate was defined as the proportion of patients who sustained a subsequent hip fracture during follow-up, whereas overall refracture rate was defined as the proportion of patients who sustained a subsequent fracture at any site during follow-up. Refracture rates at different anatomical sites and follow-up time points were extracted and analyzed separately rather than pooled, to reduce the influence of differences in outcome definitions and follow-up durations.

For BMD, HHS, and VAS outcomes, data at the final follow-up time point were extracted. BMD was restricted to measurements at the hip. Areal BMD was reported in g/cm^2^ and BMD-derived T-scores were extracted and analyzed separately. BMD was analyzed as the change from baseline to final follow-up, whereas HHS and VAS were analyzed using final follow-up values. If BMD change was not directly reported, it was calculated from the reported baseline value, final follow-up value, or percentage change. If the standard deviation of BMD change was not directly reported, it was estimated using the formula recommended by Cochrane, with the correlation coefficient between baseline and final follow-up values set at r=0.5.

### Risk of bias and methodological quality assessment

Two researchers independently assessed the risk of bias and methodological quality of the included studies. Randomized controlled trials were assessed using the Cochrane risk-of-bias tool, which evaluates random sequence generation, allocation concealment, blinding of participants and personnel, blinding of outcome assessment, incomplete outcome data, selective reporting, and other sources of bias (26). Judgments were classified as low risk, unclear risk, or high risk. Non-randomized studies were assessed using the Risk of Bias in Non-randomized Studies of Interventions (ROBINS-I) tool, which evaluates bias due to confounding, selection of participants, classification of interventions, deviations from intended interventions, missing data, measurement of outcomes, and selective reporting (27). In addition, the Methodological Index for Non-Randomized Studies (MINORS) scale was used as a supplementary assessment of methodological quality in non-randomized studies (28). The MINORS scoring criteria are shown in **Supplementary Table 2**. The scale includes 12 items, each scored as 0, 1, or 2, indicating not reported, reported but inadequate, and reported adequately and appropriately, respectively. The total score is 24, with a higher score indicating better methodological quality. Disagreements between the two researchers were resolved through discussion or, when necessary, by consultation with a third researcher.

The certainty of evidence for each outcome was assessed using the Grading of Recommendations Assessment, Development and Evaluation (GRADE) approach, considering risk of bias, inconsistency, indirectness, imprecision, and publication bias. The certainty of evidence was rated as high, moderate, low, or very low.

### Outcomes

The primary outcomes of this study included postoperative refracture-related outcomes and BMD. Refracture-related outcomes included 12-month hip refracture rate, 12-month overall refracture rate, and 24-month overall refracture rate. BMD included measurements expressed in g/cm^2^ and BMD T-score. Secondary outcomes included HHS and VAS. For refracture rate and VAS, lower values indicated better outcomes; for BMD and HHS, higher values indicated better outcomes.

### Statistical analysis

Statistical analyses were performed using R (Version 4.5.2). Bayesian network meta-analysis was mainly conducted using the gemtc and rjags packages in R, while frequentist network meta-analysis, heterogeneity analysis, and sensitivity analysis were supported by the netmeta package. Dichotomous outcomes were expressed as odds ratios (ORs) with 95% credible intervals (95% CrIs), and continuous outcomes were expressed as mean differences (MDs) with 95% CrIs. Because BMD in the included studies was reported as both g/cm^2^ and T-score, which differ in measurement unit and clinical meaning, BMD (g/cm^2^) and BMD (T-score) were analyzed separately in the network meta-analysis and were not directly pooled. When randomized controlled trials alone formed a connected treatment network, an RCT-only network meta-analysis was conducted separately. If a connected network could be formed at a specific follow-up time point, data from that time point were analyzed separately.

Before network meta-analysis, the comparability of included studies was assessed in terms of patient characteristics, interventions, follow-up durations, and outcome definitions. A Bayesian random-effects consistency model was used to synthesise direct and indirect evidence, and network plots, league tables for pairwise comparisons, and cumulative ranking probability plots were generated. Effect estimates with 95% credible intervals were considered the primary basis for interpretation, whereas ranking probabilities were treated as secondary exploratory summaries.Cumulative rankings were interpreted according to outcome direction: lower values indicated better outcomes for refracture rate and VAS, whereas higher values indicated better outcomes for BMD and HHS.

Bayesian network meta-analyses were conducted using random-effects consistency models with four Markov chain Monte Carlo chains. The adaptation iterations ranged from 5,000 to 50,000, and the simulation iterations ranged from 20,000 to 100,000, according to model requirements. Vague normal priors centered at 0 were assigned to the basic treatment effects, and a vague uniform prior was assigned to the between-study heterogeneity standard deviation in the primary models. Two additional prior specifications were fitted, consisting of Normal(0, 10^2^) priors for the basic treatment effects with Uniform(0, 2) priors for the between-study heterogeneity standard deviation, and Normal(0, 5^2^) priors with Uniform(0, 1) priors, respectively. Model convergence was assessed using the potential scale reduction factor (PSRF), effective sample size (ESS), and visual inspection of trace and posterior density plots for each outcome-specific model. Trace plots were examined to assess mixing across the four chains, whereas posterior density plots were used to examine the posterior distributions of the monitored parameters. A PSRF close to 1.00 and an adequate ESS were considered indicative of satisfactory convergence. Refracture counts were modeled using a binomial likelihood on the log-odds scale, and posterior treatment contrasts were exponentiated to obtain odds ratios.Zero-event arms were modeled directly using the binomial likelihood without applying a continuity correction.

For outcomes with closed loops, node-splitting analysis was used to evaluate consistency between direct and indirect evidence. A P value <0.05 was considered to indicate statistical inconsistency. When inconsistency was detected, data extraction and between-study differences were further checked, and the corresponding results were interpreted cautiously.

Heterogeneity among included studies was assessed using heterogeneity analysis, primarily based on the I^2^ statistic. When I^2^ was ≥50% for an outcome, indicating substantial heterogeneity, a leave-one-out sensitivity analysis was further performed to examine the influence of individual studies on the pooled effect, heterogeneity, and cumulative ranking probabilities. The I^2^ results were used to assist in judging between-study differences and were not used as a sole basis for study exclusion. Given the limited number of included studies for each outcome, comparison-adjusted funnel plots were not used to assess publication bias.

## Results

### Study selection and basic characteristics of included studies

After searching PubMed, Embase, Web of Science, and the Cochrane Library, 18 studies involving 2,414 patients with hip fractures were finally included (29–46). The study selection process is shown in **Figure 1**.

**Figure 1:**
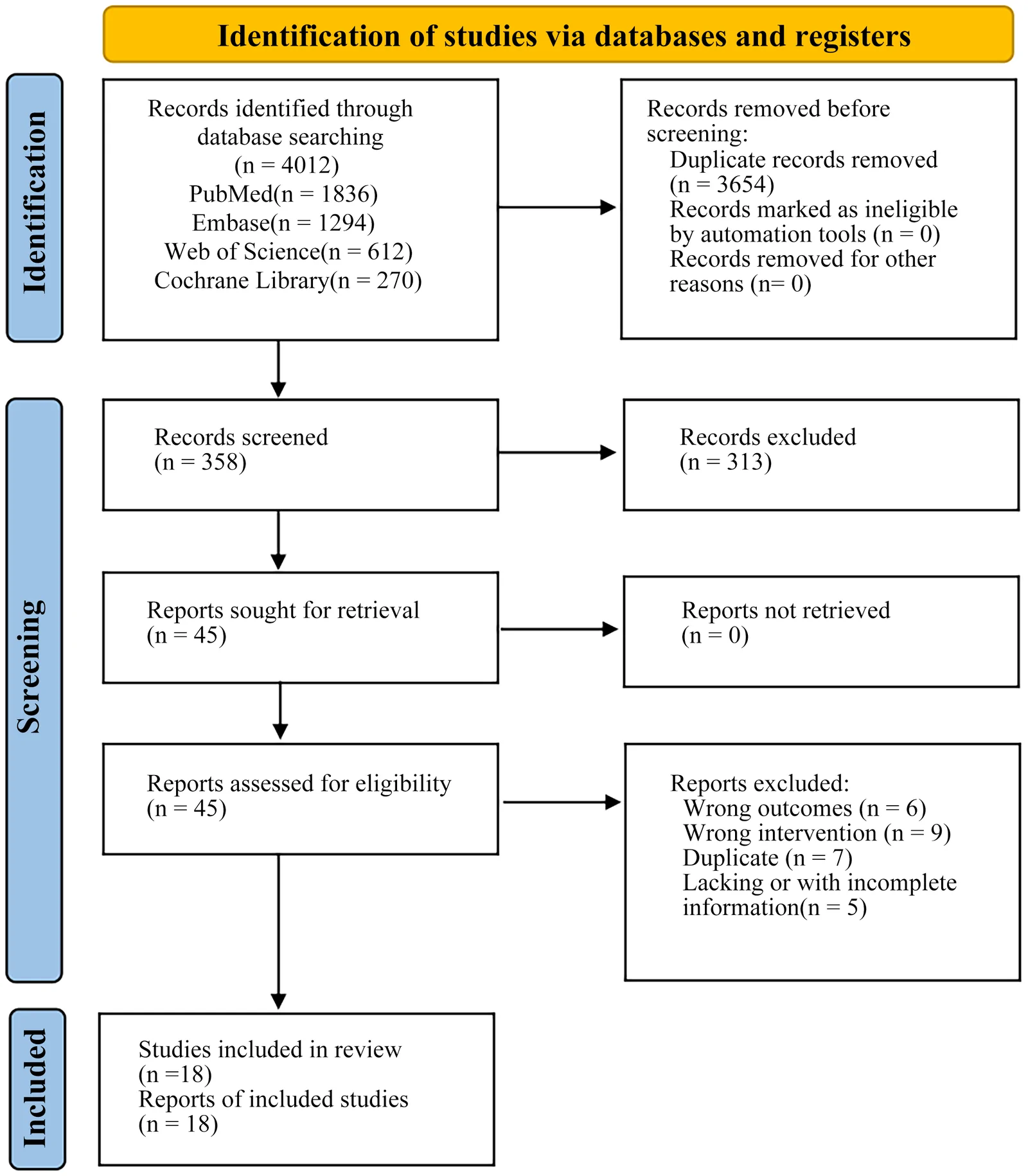
Flowchart of study selection and design.

The included studies were published between 2016 and 2025 and comprised 7 randomized controlled trials and 11 non-randomized studies. The interventions included ZOL, DEN, TPTD, ALN, RIS, ALN+TPTD, and CTRL.The included studies were characterized by baseline patient characteristics, surgical procedures, interventions, comparators, and outcome measures, as summarized in **Table 1**. Additional information on fracture type, baseline BMD or osteoporosis severity, treatment-initiation timing, follow-up duration, and calcium/vitamin D supplementation is provided in **Supplementary Table 3**.

**Table 1:** The characteristic table of the included articles.

Study and year	Country	Design	Number of patients	Surgery type	Demographic			Intervention			Outcome measures
					Treatment group 1 n (male/female)	Treatment group 2 n (male/female)	Control group n(male/female)	Treatment group 1	Treatment group 2	Control group	
Li Y, et al. (2016) (35)	China	Randomized controlled trial	60	IM	30(11/19) mean 74.99 ± 4.81 years		30(13/17) mean 73.96 ± 5.80 years	ZOL		CTRL	Healing time, BMD, VAS, HHS, Refracture rate, AE, OQOLS
Liu Z, et al. (2019) (46)	China	Prospective cohort study	482	IM	353(189/164) mean 75.41 ± 12.54 years		129(NA) mean 73.25 ± 13.75 years	ZOL		CTRL	VAS, BMD, Refracture rate, BM markers, SF-36
Koutalos AA, et al. (2022) (34)	Greece	Prospective cohort study	91	ARTH/IM	45(9/36) mean 83.6 ± 7.9 years		46(14/32) mean 79.3 ± 7.5 years	ZOL		CTRL	Mortality, Barthel, VAS, Complication
Kim SJ, et al. (2022) (41)	Korea	Retrospective cohort study	282	IM	162(16/146) mean 82.3 ± 8.1 years		120(8/112) mean 80.2 ± 7.6 years	DEN		ZOL	BMD, Persistence, Refracture rate, AE
Huang TW, et al. (2016) (36)	Taiwan	Retrospective cohort study	189	EM	47(18/29) mean 82 ± 10 years	59(21/38) mean 81 ± 8 years	83(22/61) mean 81 ± 8 years	TPTD	ALN+TPTD	CTRL	Healing time, SF-12, Pain, Refracture, BMD
Kim SJ, et al. (2018) (33)	Korea	Retrospective comparative study	96	IM	46(21/25) mean 81.6 ± 8.1 years		50(23/27) mean 82.3 ± 8.1 years	TPTD		CTRL	HHS, VAS, Healing time, Complication
Kim SJ, et al. (2019) (40)	Korea	Retrospective comparative study	112	IM	52(20/32) mean 81.4 ± 7.9 years		60(22/38) mean 80.2 ± 7.8 years	TPTD		CTRL	HHS, VAS, Healing time, Complication
Hosseini Khameneh M, et al. (2023) (37)	Iran	Randomized controlled trial	72	EM	40(18/22) mean 69.70 ± 9.0 years		32(12/20) mean 63.56 ± 5.0 years	TPTD		CTRL	HHS, VAS, Radiographic healing, Reduction failure
Tanavalee C, et al. (2025) (29)	Thailand	Randomized controlled trial	50	IM	25(7/18) mean 72.9 ± 7.5 years		25(7/18) mean 72.6 ± 7.9 years	TPTD		CTRL	Healing time, HHS, TUGT, 5xSST, BMD, Serum Ca, AE
Lee SY, et al. (2023) (39)	Korea	Randomized controlled trial	92	IM	51(all Female) mean 76.8 ± 8.32 years		41(all Female) mean 75.0 ± 8.02 years	TPTD		CTRL	CTx, OC, BMD, RUSH, Koval
Sheng OC, et al. (2024) (38)	Taiwan	Retrospective cohort study	126	IM	68(14/54) mean 85.8 ± 9.2 years		58(6/52) mean 85.0 ± 9.5 years	TPTD		DEN	Refracture rate, Healing time, VAS, Barthel, BMD
Lu X, et al. (2025) (44)	China	Retrospective comparative study	76	IM	38(10/28) mean 74.2 ± 5.9 years		38(11/27) mean 75.7 ± 4.5 years	DEN		CTRL	Healing time, Refracture rate, VAS, HHS, BMD
Wang Y, et al. (2025) (45)	China	Retrospective observational study	182	EM/ARTH	91(7/84) mean 79 ± 8.89 years		91(7/84) mean 79 ± 8.89 years	DEN		CTRL	Time to 2nd hip Fx, Refracture rate, Mortality, Cost
Wang KM, et al. (2022) (32)	China	Retrospective study	126	IM	63(34/29) age <60/≥60: 20/43		63(38/25) age <60/≥60: 19/44	ALN		CTRL	Healing time, Refracture rate, BMD, HHS, Complication
Huang TW, et al. (2016) (36)	Taiwan	Retrospective cohort study	92	ARTH	40(NA) mean 72.1 ± 7.6 years		52(NA) mean 71.0 ± 8.3 years	TPTD		CTRL	Stem subsidence, HHS, SF-12, Complication, BMD, Refracture rate, Mortality
Rana A, et al. (2021) (30)	India	Randomized controlled trial	30	IM	15(6/9) mean 74.87 ± 9.33years		15(5/10) mean 72.33 ± 9.92years	TPTD		CTRL	Healing time, LEFS, BMD, Varus collapse, Screw migration
Aggarwal HO, et al. (2025) (43)	India	Randomized controlled trial	40	IM	20(9/11) mean 69.52 ± 8.16years		20(6/14) mean 67.95 ± 10.44years	TPTD		CTRL	Healing time, BMD, RUSH, P1NP, β-CTX, Ca, PO_4_, ALP
Malouf-Sierra J, et al. (2017) (31)	Multi-country	Randomized controlled trial	216	IM/EM	106(24/82) mean 77.2 ± 8.0 years		110(25/85) mean 76.4 ± 7.5 years	TPTD		RIS	BMD, TUG, SF-36

IM, Intramedullary fixation; EM, Extramedullary fixation; ARTH, Arthroplasty; ZOL, Zoledronic acid; CTRL, Background therapy control (Ca/VitD ± placebo); DEN, Denosumab; TPTD, Teriparatide; ALN, Alendronate; RIS, Risedronate; ALN+TPTD, pre-fracture alendronate exposure followed by postoperative teriparatide treatment; BMD, Bone mineral density; VAS, Visual analogue scale; AE, Adverse events; OQOLS, Osteoporosis Quality of Life Scale; SF-36, Short Form 36; SF-12, Short Form 12; RUSH, Radiographic Union Scale for Hip; MHHS, Modified Harris Hip Score; HHS, Harris Hip Score; TUG, Timed Up and Go Test; TUGT, Timed Up and Go Test; 5xSST, Five Times Sit-to-Stand Test; CTx, C-terminal telopeptide of type I collagen; OC, Osteocalcin; Koval, Koval walking ability grading;NA, Not Applicable.

The primary outcomes of this study were postoperative refracture-related outcomes and BMD, including 12-month hip refracture rate, 12-month overall refracture rate, 24-month overall refracture rate, BMD (g/cm^2^), and BMD (T-score). Secondary outcomes included HHS and VAS. The evidence network structures for each outcome are shown in **Figure 2**. Among the outcome-specific evidence networks, closed loops were identified only for 24-month overall refracture, BMD (T-score), and VAS. Node-splitting analyses were therefore conducted only for these three outcomes.

**Figure 2:**
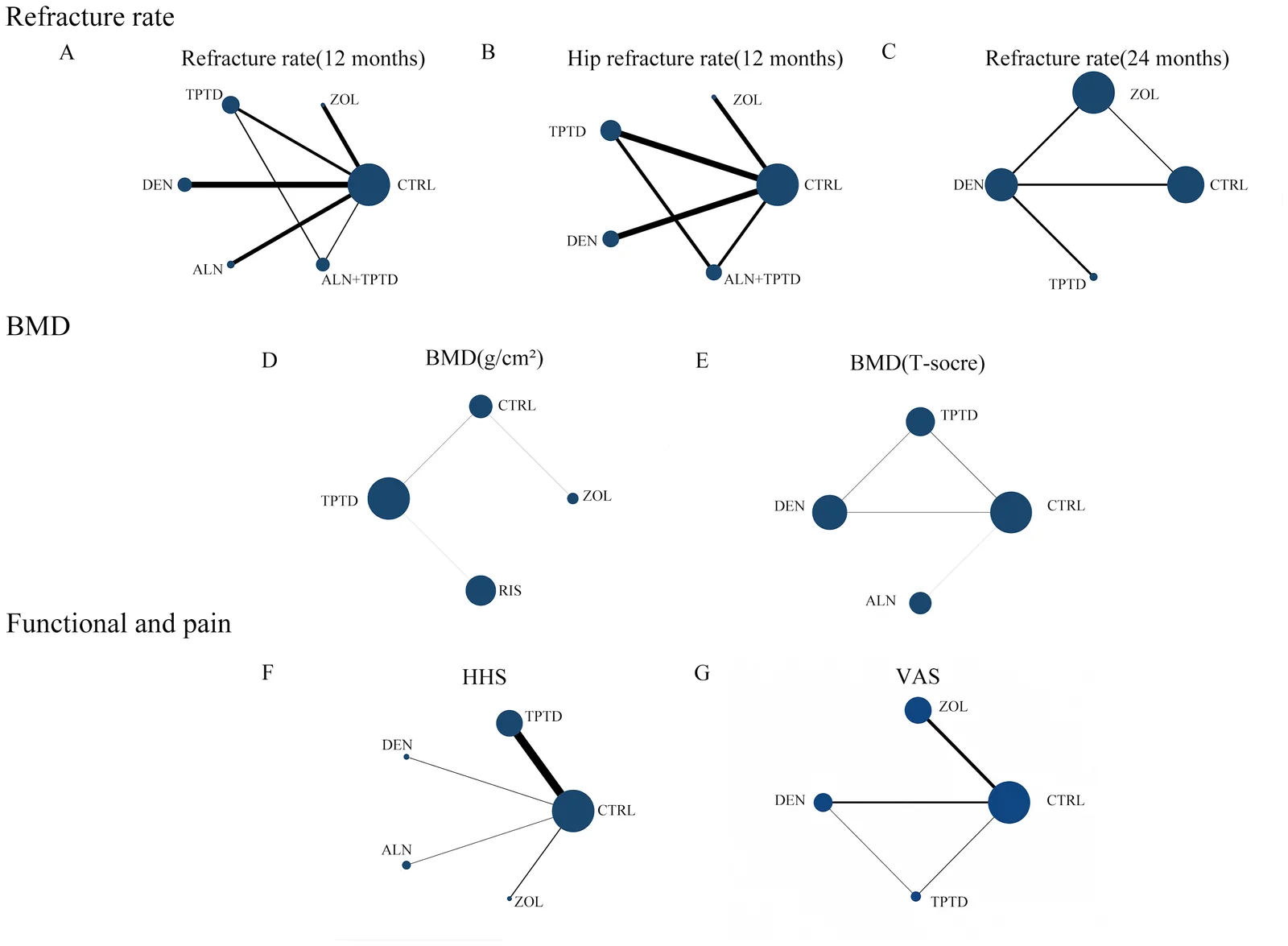
Evidence network plots for all outcomes. **(A)** 12-month overall refracture rate; **(B)** 12-month hip refracture rate; **(C)** 24-month overall refracture rate; **(D)** hip bone mineral density in g/cm^2^; **(E)** hip bone mineral density T-score; **(F)** Harris Hip Score; **(G)** visual analog scale score. Node size represents the sample size of each intervention, and line thickness represents the amount of direct comparative evidence. ALN, alendronate; ALN+TPTD, pre-fracture alendronate exposure followed by postoperative teriparatide treatment; BMD, bone mineral density; CTRL, background therapy control (Ca/VitD ± placebo); DEN, denosumab; HHS, Harris Hip Score; RIS, risedronate; TPTD, teriparatide; VAS, visual analog scale; ZOL, zoledronic acid.

### Convergence diagnostics

Convergence diagnostics were assessed for all seven outcome-specific Bayesian models. The maximum PSRF/minimum ESS values were 1.065/177.75 for 12-month overall refracture, 1.009/295.17 for 12-month hip refracture, 1.002/5,848.20 for 24-month overall refracture, 1.000/41,405.05 for HHS, 1.000/29,502.86 for VAS, 1.000/11,233.76 for BMD (g/cm^2^), and 1.000/23,764.93 for BMD (T-score). The 12-month refracture models showed comparatively higher PSRF values and lower ESS, consistent with their sparse comparisons and zero-event data. Trace and posterior density plots are presented in **Supplementary Material 3** (**Figures S1**–**S7**), and the complete numerical convergence diagnostics are reported in **Supplementary Table 4**.

### Risk of bias and methodological quality assessment

The ROBINS-I risk-of-bias assessment results for non-randomized studies are shown in **Figure 3A**, the Cochrane risk-of-bias assessment results for randomized controlled trials are shown in **Figure 3B**, and the MINORS methodological quality scores for non-randomized studies are shown in **Table 2**. Detailed ROBINS-I assessment results for non-randomized studies are shown in **Supplementary Table 5**. Based on the ROBINS-I tool, all 11 non-randomized studies were judged to have a high overall risk of bias, with the main concerns involving participant selection, classification of interventions, deviations from intended interventions, outcome measurement, and selection of reported results. MINORS scores ranged from 16 to 21, suggesting that the overall methodological quality of the included non-randomized studies was moderate to good. Most studies clearly stated their objectives, used outcomes consistent with their objectives, and applied appropriate statistical methods for between-group comparisons. The main limitations were related to prospective data collection, sample size calculation, reporting of loss to follow-up, and blinding of outcome assessment. Among the 7 randomized controlled trials, some studies had unclear or high risk of bias in random sequence generation, allocation concealment, and blinding, whereas the overall risk was low for completeness of outcome data and selective reporting.

**Figure 3:**
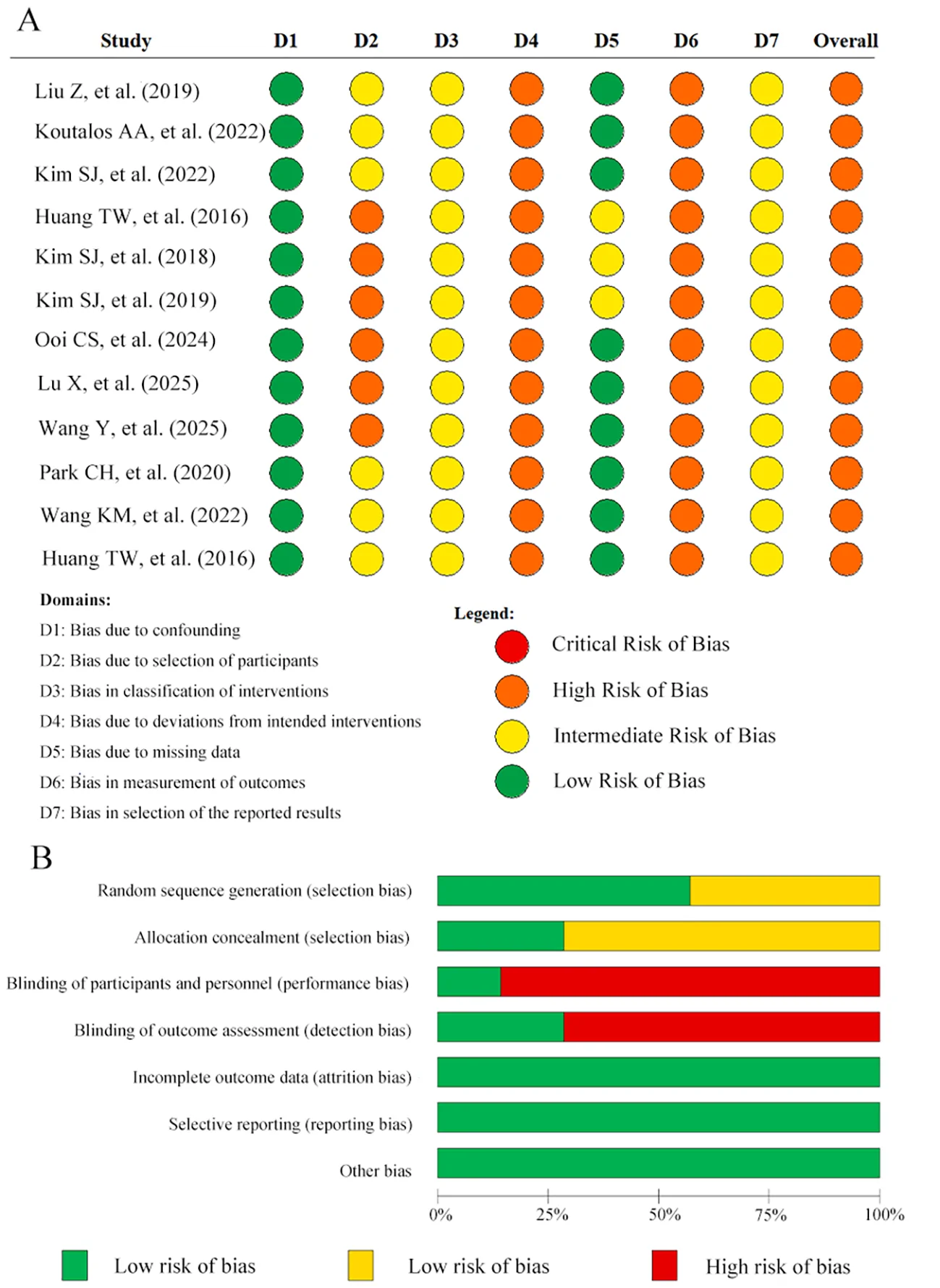
Risk-of-bias assessment of the included studies. **(A)** ROBINS-I assessment for non-randomized studies; **(B)** Cochrane risk-of-bias assessment for randomized controlled trials. D1–D7 indicate the corresponding domains of the risk-of-bias tools.

**Table 2:** Methodological quality of the studies (MINORS).

Study	A	B	C	D	E	F	G	H	I	J	K	L	Total scores
Liu 2019 (46)	2	1	1	2	1	2	2	0	2	2	2	1	18
Koutalos 2021 (34)	2	2	2	2	1	2	1	2	2	2	1	2	21
Kim 2018 (33)	2	1	0	2	2	2	1	0	2	2	2	2	18
Huang 2016 3 (36)	2	1	0	2	1	2	1	0	2	2	2	2	17
Kim 2022 (41)	2	1	0	2	1	2	1	0	2	2	1	2	16
Kim 2019 (40)	2	1	0	2	2	2	1	0	2	2	2	2	18
Sheng 2024	2	1	0	2	1	2	1	0	2	2	2	1	16
Lu 2025 (44)	2	1	0	2	1	2	1	0	2	2	2	2	17
Y. Wang 2025 (45)	2	1	0	2	1	2	1	0	2	2	2	2	17
K. Wang 2022 (32)	2	1	0	2	2	2	1	0	2	2	2	2	18
Huang 2016 (36)	2	1	0	2	2	2	2	0	2	2	2	2	19

### Hip refracture rate (12 months)

Five studies involving 599 patients provided data on the 12-month hip refracture rate, including five interventions: ZOL, TPTD, DEN, ALN+TPTD, and CTRL (35, 36, 42, 44, 45). The network structure for this outcome is shown in **Figure 2B**; pairwise comparison results are shown in **Figure 4A** (upper triangle), and the cumulative ranking probabilities are shown in **Figure 4D**.

**Figure 4:**
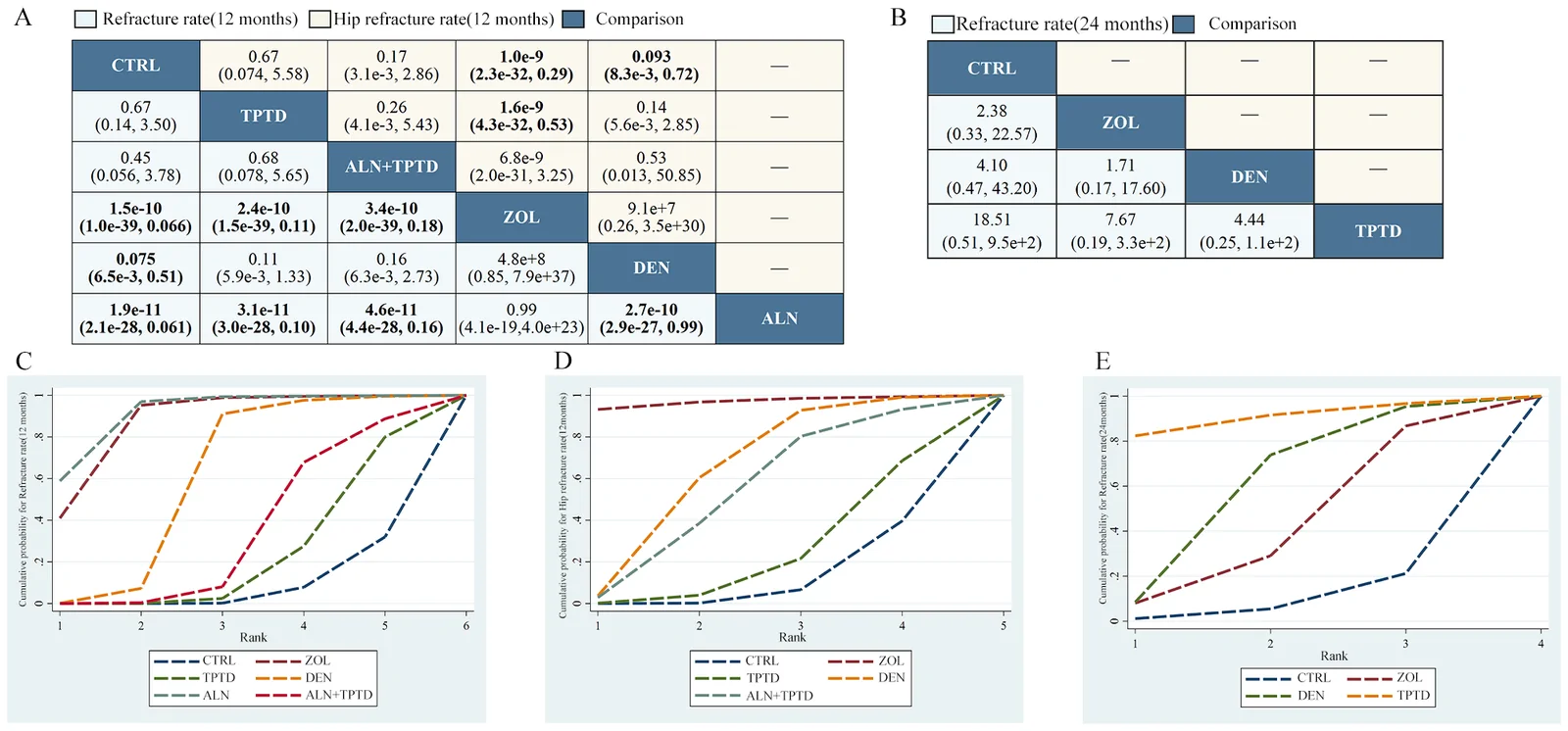
Network meta-analysis results and cumulative ranking probabilities for refracture outcomes. **(A)** League table for 12-month overall refracture rate and 12-month hip refracture rate; **(B)** league table for 24-month overall refracture rate; **(C)** cumulative ranking probabilities for 12-month overall refracture rate; **(D)** cumulative ranking probabilities for 12-month hip refracture rate; **(E)** cumulative ranking probabilities for 24-month overall refracture rate. Odds ratios with 95% credible intervals are shown in the league tables. ALN, alendronate; ALN+TPTD, pre-fracture alendronate exposure followed by postoperative teriparatide treatment; CrI, credible interval; CTRL, background therapy control (Ca/VitD ± placebo); DEN, denosumab; OR, odds ratio; TPTD, teriparatide; ZOL, zoledronic acid.

For the 12-month hip refracture rate, ZOL showed a trend toward reduced risk compared with CTRL (OR: 1.0e-9 [2.3e-32, 0.29]), and DEN also showed a trend toward reduced risk compared with CTRL (OR: 0.093 [8.3e-3, 0.72]). In addition, ZOL showed a trend toward reduced risk compared with TPTD (OR: 1.6e-9 [4.3e-32, 0.53]). Most other comparisons had 95% CrIs crossing the null line, and no clear between-intervention differences were observed.

Given the small number of events for this outcome, the presence of zero events in some comparisons, and limited direct head-to-head evidence, these findings should be interpreted as trends suggesting risk reduction rather than definitive differences in efficacy.

Heterogeneity for this outcome was overall low, and detailed results are shown in **Supplementary Table 6**. Cumulative ranking probabilities for all interventions are provided in **Supplementary Table 7** for descriptive reference. Leave-one-out sensitivity analysis results are shown in **Supplementary Table 8**, with the overall findings remaining broadly stable.

### Postoperative overall refracture rate (12 and 24 months)

Postoperative overall refracture rate included two follow-up time points: 12 months and 24 months. For the 12-month overall refracture rate, the network structure is shown in **Figure 2A**, pairwise comparison results are shown in **Figure 4A** (lower triangle), and cumulative ranking probabilities are shown in **Figure 4C**. For the 24-month overall refracture rate, the network structure is shown in **Figure 2C**, pairwise comparison results are shown in **Figure 4B**, and cumulative ranking probabilities are shown in **Figure 4E**. Six studies involving 725 patients provided data on the 12-month overall refracture rate, covering six interventions: ZOL, TPTD, DEN, ALN, ALN+TPTD, and CTRL (32, 35, 36, 42, 44, 45). Four studies involving 1,072 patients provided data on the 24-month overall refracture rate, covering four interventions: ZOL, DEN, TPTD, and CTRL (38, 41, 45, 46).

For the 12-month overall refracture rate, ZOL-related comparisons generally showed trends toward reduced risk: ZOL versus CTRL (OR: 1.5e-10 [1.0e-39, 0.066]), TPTD (OR: 2.4e-10 [1.5e-39, 0.11]), and ALN+TPTD (OR: 3.4e-10 [2.0e-39, 0.18]) all suggested lower refracture risk. DEN also showed a trend toward reduced risk compared with CTRL (OR: 0.075 [0.0065, 0.51]). ALN-related comparisons similarly showed trends toward reduced risk, including ALN versus CTRL (OR: 1.9e-11 [2.1e-28, 0.061]), TPTD (OR: 3.1e-11 [3.0e-28, 0.10]), ALN+TPTD (OR: 4.6e-11 [4.4e-28, 0.16]), and DEN (OR: 2.7e-10 [2.9e-27, 0.99]). The 95% CrIs for all other comparisons crossed the null line, and no clear between-intervention differences were observed. For the 24-month overall refracture rate, no clear differences were observed among interventions.

Given the small number of postoperative overall refracture events, the presence of zero events in some comparisons, limited direct head-to-head evidence between relevant interventions, and the occurrence of extreme effect estimates with wide credible intervals in some comparisons, these risk-reduction trends and ranking results should be interpreted cautiously as trend-based findings rather than definitive judgments of comparative intervention superiority.

In terms of heterogeneity, heterogeneity for the 12-month overall refracture rate was overall low, whereas heterogeneity for the 24-month overall refracture rate was low to moderate. Detailed results are shown in **Supplementary Tables 10** and **11**, respectively. For the 24-month overall refracture network, node-splitting analyses showed no evidence of inconsistency between direct and indirect estimates across the three comparisons (P = 0.27–0.28). The corresponding direct, indirect, and network estimates with 95% CrIs are presented in **Figure 5A** and **Supplementary Table 12**. Cumulative ranking probabilities for the 12- and 24-month overall refracture outcomes are provided in **Supplementary Tables 13** and **14**, respectively, for descriptive reference. Leave-one-out sensitivity analysis results are shown in **Supplementary Tables 9** and **15**, respectively, with the overall findings remaining broadly stable at both follow-up time points.

**Figure 5:**
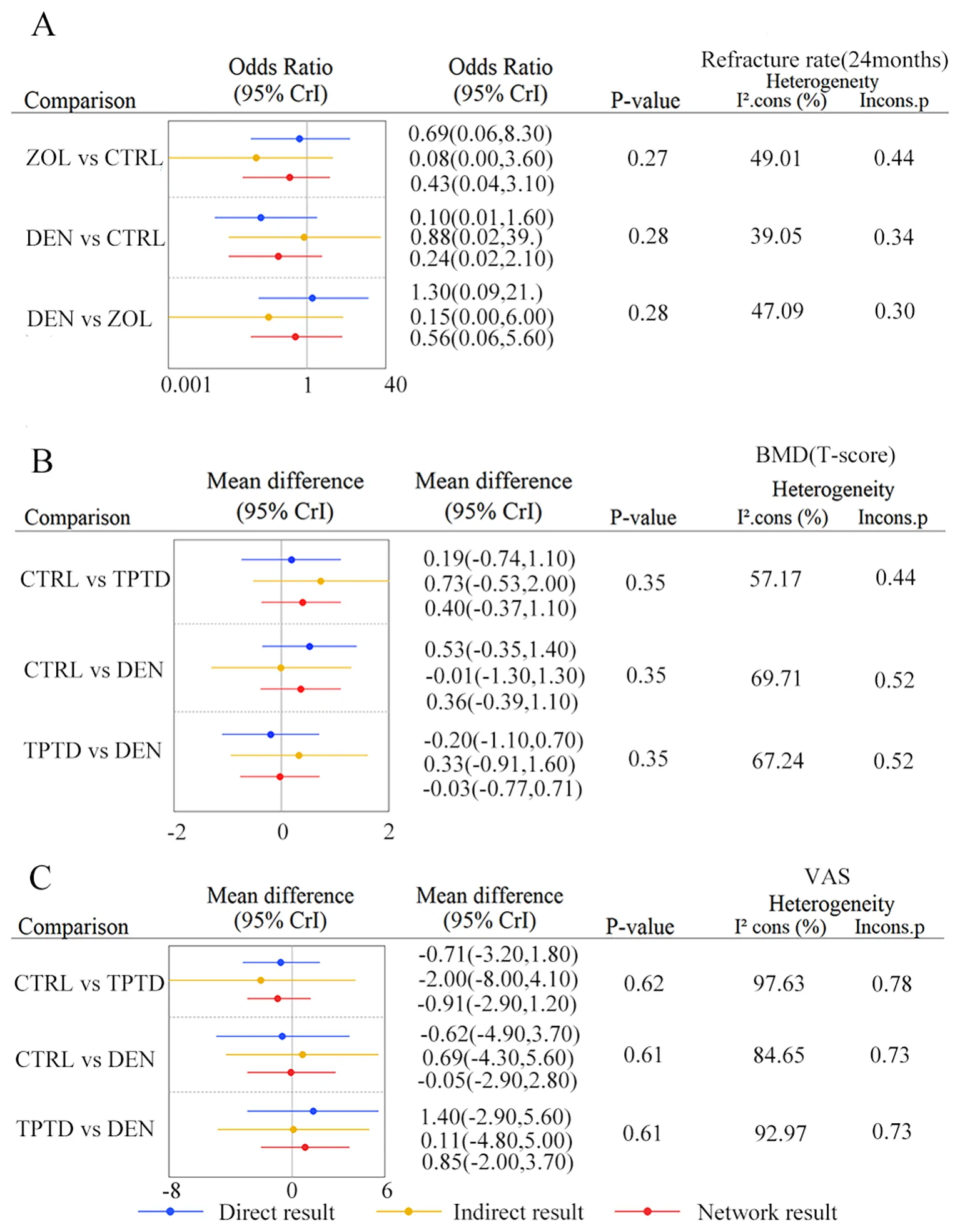
Node-splitting analysis for outcomes with closed loops. **(A)** 24-month overall refracture rate; **(B)** hip bone mineral density T-score; **(C)** visual analog scale score. Direct, indirect, and network estimates are presented with 95% credible intervals, together with inconsistency P values and heterogeneity statistics. ALN, alendronate; BMD, bone mineral density; CrI, credible interval; CTRL, background therapy control (Ca/VitD ± placebo); DEN, denosumab; MD, mean difference; OR, odds ratio; TPTD, teriparatide; VAS, visual analog scale; ZOL, zoledronic acid.

Several refracture comparisons involving sparse or zero-event data yielded extreme ORs. Alternative prior specifications changed the magnitude of some estimates, while the corresponding 95% CrIs remained wide (**Supplementary Table 16**). Observed refracture events and absolute event rates for each intervention and follow-up time point are provided in **Supplementary Tables 46**–**48**.

### BMD

BMD-related outcomes included BMD (g/cm^2^) and BMD (T-score). The network structures for BMD (g/cm^2^) and BMD (T-score) are shown in **Figure 2D** and **Figure 2E**, respectively. Pairwise comparison results for the two BMD outcomes are shown in **Figure 6A**. The cumulative ranking probabilities for BMD (g/cm^2^) and BMD (T-score) are shown in **Figure 6B** and **Figure 6C**, respectively.

**Figure 6:**
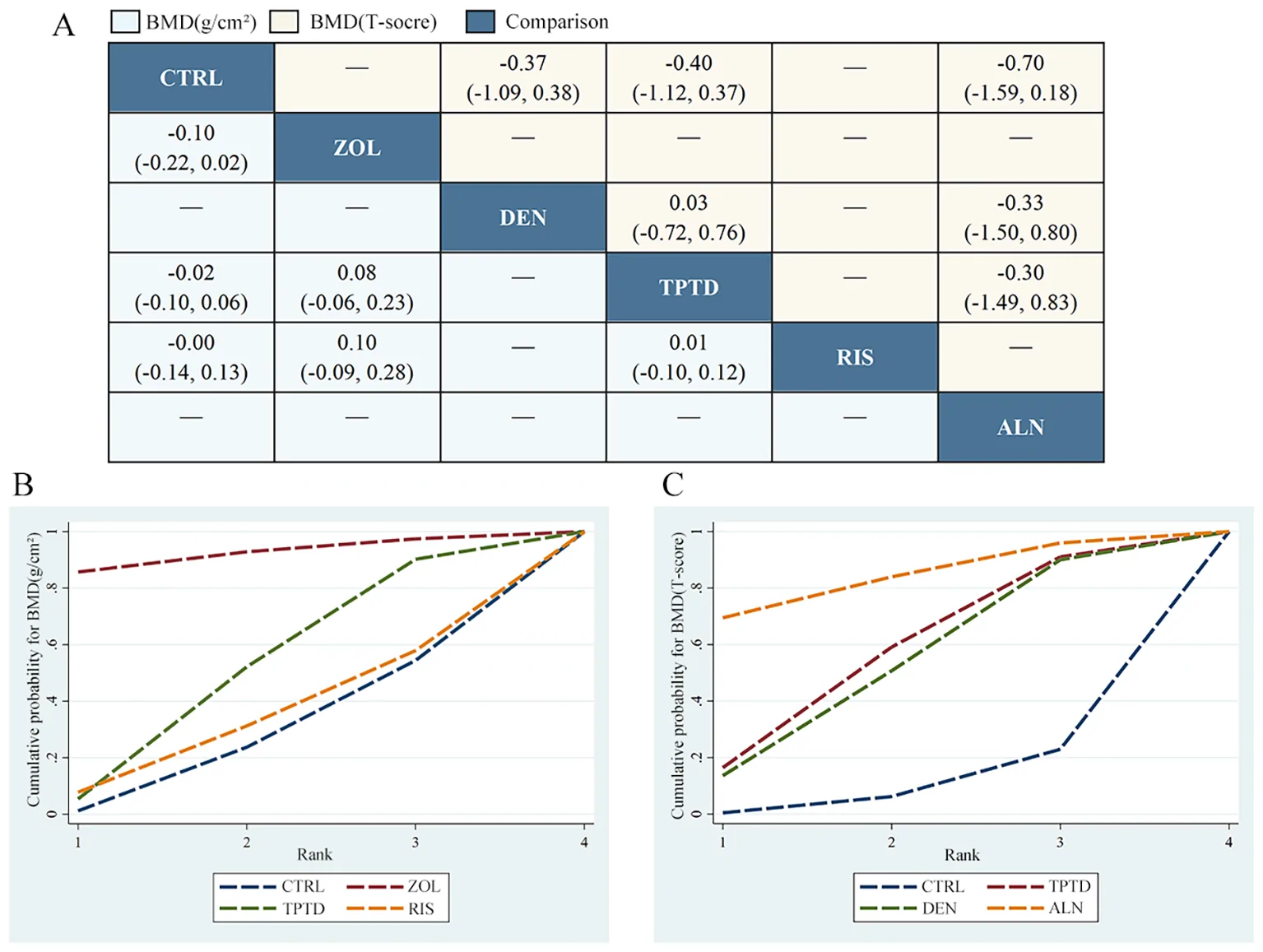
Network meta-analysis results and cumulative ranking probabilities for hip bone mineral density outcomes. **(A)** League table for hip bone mineral density expressed in g/cm^2^ and hip bone mineral density T-score; **(B)** cumulative ranking probabilities for hip bone mineral density in g/cm^2^; **(C)** cumulative ranking probabilities for hip bone mineral density T-score. Mean differences with 95% credible intervals are shown in the league table. ALN, alendronate; BMD, bone mineral density; CrI, credible interval; CTRL, background therapy control (Ca/VitD ± placebo); DEN, denosumab; MD, mean difference; RIS, risedronate; TPTD, teriparatide; ZOL, zoledronic acid.

For BMD (g/cm^2^), four studies involving 301 patients provided BMD data, covering four interventions: ZOL, TPTD, RIS, and CTRL (30, 31, 35, 43). All studies contributing to hip BMD (g/cm^2^) were RCTs; therefore, the corresponding network was based exclusively on randomized evidence. For BMD (T-score), four studies involving 368 patients provided BMD data, covering four interventions: TPTD, DEN, ALN, and CTRL (32, 38, 43, 44). Across the BMD-contributing studies, the reported hip sites, laterality, DXA methods/devices, and follow-up time points varied, as shown in **Supplementary Table 3**, Panel B. No clear between-intervention differences were observed for either BMD outcome.

In terms of heterogeneity, heterogeneity for BMD (g/cm^2^) was overall low, whereas heterogeneity for BMD (T-score) was moderate. Detailed results are shown in **Supplementary Tables 17** and **18**, respectively. For the BMD (T-score) network, node-splitting analyses showed no evidence of inconsistency between direct and indirect estimates across the three comparisons (P = 0.35 for all comparisons). The corresponding direct, indirect, and network estimates with 95% CrIs are presented in **Figure 5B** and **Supplementary Table 19**.

Cumulative ranking probabilities for BMD (g/cm^2^) and BMD (T-score) are presented in **Figure 6B** and **Figure 6C** and **Supplementary Tables 20** and **21**, respectively, for descriptive reference. Leave-one-out sensitivity analysis results are shown in **Supplementary Tables 23** and **24**, respectively, with the overall findings remaining broadly stable for both BMD measures.

### HHS

Eight studies involving 684 patients provided HHS data, covering five interventions: ZOL, DEN, TPTD, ALN, and CTRL (29, 32, 33, 35, 37, 40, 42, 44). The network structure for this outcome is shown in **Figure 2F**. No clear between-intervention differences were observed, and pairwise comparison results with the corresponding effect estimates and 95% CrIs are shown in **Figure 7A** (HHS section). Regarding heterogeneity, the I^2^estimate for the HHS outcome was 97.25%, indicating high between-study heterogeneity. The remaining detailed heterogeneity results are presented in **Supplementary Table 25**. Cumulative ranking probabilities for HHS are shown in **Figure 7B** and **Supplementary Table 26** for descriptive reference.

**Figure 7:**
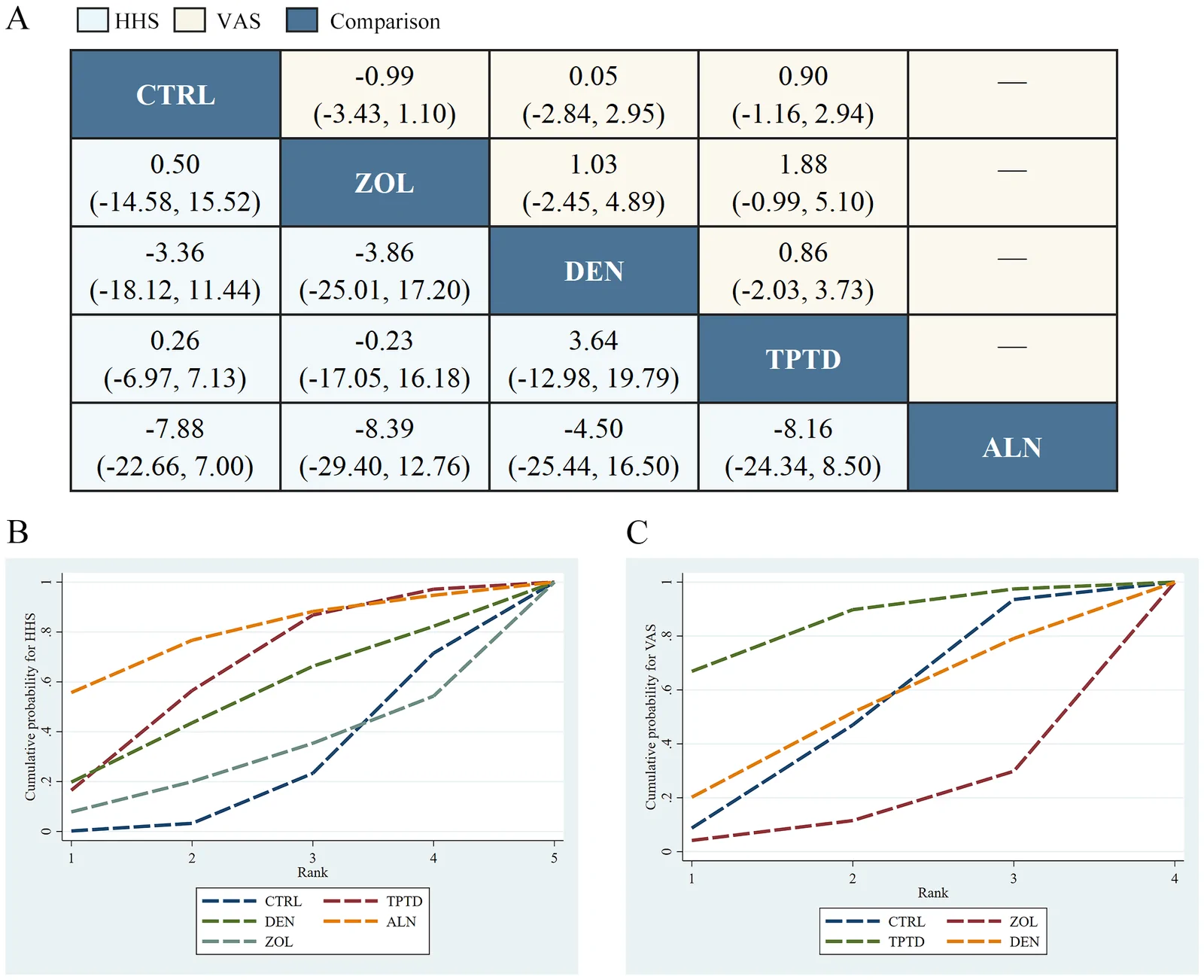
Network meta-analysis results and cumulative ranking probabilities for functional and pain outcomes. **(A)** League table for Harris Hip Score and visual analog scale score; **(B)** cumulative ranking probabilities for Harris Hip Score; **(C)** cumulative ranking probabilities for visual analog scale score. Mean differences with 95% credible intervals are shown in the league table. ALN, alendronate; CrI, credible interval; CTRL, background therapy control (Ca/VitD ± placebo); DEN, denosumab; HHS, Harris Hip Score; MD, mean difference; TPTD, teriparatide; VAS, visual analog scale; ZOL, zoledronic acid.

Leave-one-out sensitivity analysis results are shown in **Supplementary Table 27**, with the overall findings remaining broadly stable. For HHS, treatment effect estimates and ranking probabilities were compared between the primary network meta-analysis and the RCT-only sensitivity analysis, and an exact-12-month follow-up sensitivity analysis was conducted. The results are shown in **Supplementary Tables 28**–**30**.

HHS reflects recovery within a broader surgical and rehabilitation pathway, rather than the effect of medication in isolation. All eight studies used the same or essentially the same surgical procedure or fixation device within each medication comparison, improving within-study comparability. Nevertheless, surgical procedures varied across studies and were not included in multivariable adjustment; therefore, differences in HHS cannot be primarily attributed to medication choice alone.

### VAS

Eight studies involving 1,074 patients provided VAS data, covering four interventions: ZOL, DEN, TPTD, and CTRL (33–35, 37, 38, 40, 44, 46). The network structure for this outcome is shown in **Figure 2G**. No clear between-intervention differences were observed, and pairwise comparison results with the corresponding effect estimates and 95% CrIs are shown in **Figure 7A** (VAS section). For the VAS network, node-splitting analyses showed no evidence of inconsistency between direct and indirect estimates across the three comparisons (P = 0.61–0.62). The corresponding direct, indirect, and network estimates with 95% CrIs are presented in **Figure 5C** and **Supplementary Table 31**. Regarding heterogeneity, the I^2^for the VAS outcome was 99.18%, indicating high between-study heterogeneity. The remaining detailed heterogeneity results are presented in **Supplementary Table 32**. Cumulative ranking probabilities for VAS are shown in **Figure 7C** and **Supplementary Table 33** for descriptive reference.

Leave-one-out sensitivity analysis results are shown in **Supplementary Table 34**, with the overall findings remaining broadly stable. For VAS, treatment effect estimates and ranking probabilities were compared between the primary network meta-analysis and the RCT-only sensitivity analysis, and an exact-12-month follow-up sensitivity analysis was conducted. The results are shown in **Supplementary Tables 28–30**.

VAS likewise captures the overall postoperative pain experience, rather than the effect of medication alone. Five of eight studies used the same surgical procedure or fixation device, although one included mixed procedures and two provided limited procedural details. Because no study explicitly adjusted VAS for surgical procedure, differences in VAS cannot be primarily attributed to medication choice alone.

## Discussion

In this systematic review and Bayesian network meta-analysis of 18 studies involving 2,414 patients, we found that the comparative effects of anti-osteoporosis medications after hip fracture surgery varied according to treatment goal and follow-up duration. Overall, the findings did not support a universal treatment hierarchy but provided clinically relevant signals for postoperative refracture prevention, bone health management, functional recovery, and pain improvement. Given the limited and predominantly low-certainty evidence, these findings should be interpreted cautiously and considered within the broader clinical context of postoperative care.

Compared with previous meta-analyses, which have mainly focused on postmenopausal osteoporosis, fracture prevention, bone mineral density, or selected anti-osteoporosis medications, the present study specifically examined patients after surgery for fragility hip fracture (47–50). In this population, anti-osteoporosis treatment forms part of a broader strategy for secondary fracture prevention and should be considered together with fracture liaison services, rehabilitation, and continuity of care (51–54). Previous evidence and clinical guidelines have shown that a history of low-energy or osteoporotic fracture is associated with an increased risk of subsequent fracture, and subsequent fractures may further increase mortality risk (55–58). The present study therefore provides a more focused comparison of multiple medications across refracture, BMD, hip function, and pain outcomes in the postoperative hip-fracture setting.

The refracture findings merit particular caution. Across refracture outcomes, comparative estimates varied according to refracture definition and follow-up duration, with interpretation further limited by uncommon events, zero-event comparisons, and limited direct evidence. For BMD, numerical ranking patterns varied across reporting formats, although stable between-intervention differences were not established. HHS and VAS showed patterns that were not fully consistent with refracture and BMD, highlighting the influence of surgical procedure, rehabilitation, perioperative management, and baseline patient status (59–61). Across all outcome domains, ranking probabilities were interpreted as exploratory summaries alongside the effect estimates rather than as evidence of treatment priority. The implications of these outcome-specific patterns are considered in detail below.

ZOL showed numerically favorable comparative results for both 12-month overall refracture and 12-month hip refracture outcomes, although the corresponding estimates were imprecise. Although surgery restores stability at the fracture site, the patient’s underlying osteoporosis, reduced activity, muscle weakness, and fall risk do not disappear immediately after surgery (59–61). As an intravenous bisphosphonate, ZOL can reduce ongoing bone loss by inhibiting osteoclast-mediated bone resorption (62). Previous studies have also shown that ZOL can increase BMD and reduce fracture risk, and analyses in patients after hip fracture suggest that its potential benefits may extend beyond fracture prevention (62–64). Therefore, the observed direction of the estimates is compatible with its antiresorptive mechanism and previous evidence, but should not be interpreted as evidence of comparative superiority. In addition, its once-yearly intravenous administration may be more convenient for older patients who have difficulty maintaining long-term oral therapy, have poor gastrointestinal tolerance, or have limited adherence.

DEN showed numerically favorable signals for 12-month hip refracture, 12-month overall refracture, and 24-month overall refracture, suggesting a potentially consistent pattern across follow-up time points. DEN is an antiresorptive medication with a clear mechanism of action, reducing osteoclast formation, function, and survival by inhibiting RANKL (65). Previous osteoporosis studies have also shown that DEN can reduce fracture risk and improve BMD (65, 66). However, no stable differences among interventions were established for the 24-month overall refracture rate, and direct comparative evidence between DEN and other medications remains limited. Therefore, DEN should be regarded as a candidate regimen worthy of attention rather than as clearly superior to other medications. In clinical use, continuous treatment and sequential therapy after discontinuation should be planned in advance to reduce the risk of rebound bone turnover and vertebral fractures after stopping DEN (67).

ALN showed numerically favorable estimates for the 12-month overall refracture rate, indicating that traditional oral bisphosphonate therapy remains clinically relevant in postoperative management. However, because no consistent between-intervention superiority was established, this pattern should not be interpreted as evidence that ALN is preferred to other medications. ALN mainly reduces bone loss by inhibiting bone resorption, and its effects in reducing fracture risk and maintaining BMD have been supported by previous studies and relevant guidelines (47, 68, 69). However, ALN requires strict administration procedures and good adherence. It is more suitable for patients who can take the drug correctly, have good gastrointestinal tolerance, and are able to continue treatment after discharge. In older patients with swallowing difficulties, cognitive impairment, or insufficient caregiving support, the actual benefit may be limited.

After the follow-up was extended to 24 months, the numerical patterns for TPTD and DEN appeared more favorable than those observed at earlier follow-up. However, no clear between-intervention differences were observed, and these patterns should be interpreted in the context of the sparse evidence and limited number of events. As an anabolic agent, TPTD may exert its effect more through medium- to long-term improvement in bone mass and restoration of bone strength, rather than producing immediate early postoperative benefits. Previous studies of TPTD in patients after surgery for osteoporotic hip fracture have suggested possible positive effects on fracture healing or early functional recovery (70–72). However, randomized controlled trials and systematic reviews have not yielded fully consistent results (71, 72). TPTD may therefore be considered in patients with severe osteoporosis or a greater need for bone mass recovery, although the current evidence does not establish a ranking-based treatment preference.

Across the two BMD outcomes, the numerical ordering differed between g/cm^2^ and T-score, with ZOL and TPTD showing relatively favorable numerical patterns for BMD (g/cm^2^) and ALN and TPTD for BMD (T-score), respectively. However, no clear between-intervention differences were observed for either outcome, and these patterns should be interpreted as descriptive trends rather than evidence of comparative efficacy. BMD is a commonly used monitoring indicator in osteoporosis treatment, and its improvement is associated with fracture risk reduction; nevertheless, BMD alone cannot fully explain an individual’s actual fracture risk (74, 75). The pharmacological characteristics of ZOL and ALN may provide context for the observed numerical patterns because of their antiresorptive effects and established roles in maintaining bone mass, although these considerations do not establish treatment superiority. The interpretation of these findings is further complicated by differences in BMD reporting format, baseline BMD, anatomical site and laterality, DXA methods or devices, follow-up duration, and sample size. Although standardized mean differences could accommodate different measurement scales, they would not resolve this underlying heterogeneity and might reduce clinical interpretability. Site-specific analyses were not feasible because the available studies were sparse and did not form sufficiently connected networks within a common hip subregion. Accordingly, the pooled BMD findings should be viewed as scale-specific comparative estimates across heterogeneous postoperative populations rather than as universally applicable effects across all hip regions or specific fracture and surgical pathways.

Given the characteristics of HHS and VAS outcomes, these results should be interpreted within the postoperative recovery process. Compared with refracture and BMD, hip joint function and pain are not determined solely by anti-osteoporosis medications. For HHS, ALN showed a numerically favorable pattern in the main analysis and remained relatively favorable in most leave-one-out sensitivity analyses. However, no clear between-intervention differences were observed, and heterogeneity decreased markedly after excluding Huang TW et al. (2016), indicating that the HHS outcome may still have been influenced by individual studies. HHS is a composite functional indicator that is affected not only by bone mass status but also by fracture type, surgical procedure, internal fixation or arthroplasty outcome, preoperative mobility, postoperative rehabilitation, and complications (76, 77). Therefore, the observed numerical pattern for ALN and TPTD should be interpreted as a tentative signal in the direction of functional recovery rather than as evidence that these medications reliably improve hip joint function.

For VAS, ZOL and DEN showed numerically favorable patterns, but no clear between-intervention differences were observed. Pain is an important issue during recovery after hip fracture surgery; however, VAS scores can be influenced by many factors, including surgical trauma, quality of fracture reduction, analgesic regimen, early ambulation, muscle strength recovery, and comorbidities. Therefore, pain control and functional recovery cannot be attributed simply to a single anti-osteoporosis medication, but should be understood in combination with perioperative analgesia, early rehabilitation, multidisciplinary management, and continuous care (76, 77). The observed numerical pattern for ZOL and DEN should therefore be interpreted as a tentative signal in pain-related outcomes rather than as evidence of direct analgesic effects.

Across all outcomes, leave-one-out sensitivity analyses suggested that the overall findings remained broadly stable after excluding individual studies, although some variation was observed in individual estimates and ranking positions. For HHS and VAS, separate RCT-only analyses were conducted because the available RCTs formed connected treatment networks. These analyses were not extended to the remaining outcomes because the available RCTs did not form connected networks; for hip BMD (g/cm^2^), all contributing studies were RCTs, and a separate RCT-only analysis was therefore unnecessary. Exact 12-month follow-up analyses were conducted where sufficient data were available at this standardized time point to reduce the potential influence of differences in follow-up duration. Together, these complementary analyses supported the overall stability of the findings across alternative analytical conditions. Nevertheless, uncertainty remained because of sparse treatment networks, small sample sizes, few fracture events, zero-event comparisons, between-study heterogeneity, and differences in BMD reporting formats. Accordingly, sensitivity analyses provided supportive evidence for the robustness of the findings, although treatment rankings should still be interpreted as reference signals rather than definitive evidence of treatment superiority or fixed treatment hierarchies.

Treatment-initiation timing should also be considered as a potential source of clinical heterogeneity. The interval between surgery and initiation of anti-osteoporosis therapy may influence drug exposure during the early postoperative period and the time available for BMD improvement, particularly when comparing antiresorptive and anabolic strategies. However, treatment-initiation timing was reported inconsistently across the included studies, preventing the formation of sufficiently comparable groups for an independent subgroup analysis. Thus, treatment-initiation timing may be relevant to the interpretation of these findings, but the available evidence does not allow its contribution to be disentangled from treatment class and other clinical characteristics.

From a clinical application perspective, the findings of this study are more appropriately understood as medication tendencies under different treatment goals rather than as a fixed medication priority order. Some early refracture comparisons showed relatively favorable efficacy signals for ZOL, DEN, and ALN, but these signals should be regarded as contextual considerations rather than treatment recommendations. In practice, medication selection would need to account for individual fracture risk, renal function, calcium/vitamin D status, gastrointestinal tolerance, adherence, administration burden, treatment feasibility, patient preference, and access. ZOL’s once-yearly intravenous administration may be relevant for patients who have difficulty maintaining long-term oral therapy, whereas DEN’s subcutaneous administration may be relevant when oral bisphosphonate treatment is difficult; however, ongoing treatment, discontinuation planning, and safety monitoring remain important (62–67). ALN may remain a practical oral option for patients who can tolerate and adhere to oral treatment (68, 69). For patients with more severe osteoporosis, a history of multiple fractures, or a focus on medium- to long-term bone mass recovery, TPTD may be considered as a context-dependent option rather than a preferred treatment (70, 73). Because comparative safety, treatment discontinuation, adherence, renal considerations, and hypocalcemia were not systematically evaluated, these considerations should be integrated with the broader postoperative care plan and should not be determined by ranking patterns alone.

Overall, this study provides a focused synthesis of current evidence on anti-osteoporosis medications after surgery for fragility hip fracture. Unlike previous reviews that mainly focused on postmenopausal osteoporosis or single or selected medication classes, this study specifically focused on patients after fragility hip fracture surgery, a population at high risk of secondary fractures, and included four outcome domains: refracture, BMD, hip joint function, and pain. This makes the findings more relevant to clinical decision-making under different postoperative treatment goals. Methodologically, this study used a network meta-analysis framework to integrate comparative information from different studies within the same evidence network, thereby more systematically presenting the relative performance and ranking signals of multiple clinically used anti-osteoporosis regimens across different outcomes. These results may help clinicians better understand the potential roles of different medications in postoperative secondary fracture prevention and individualised treatment selection.

From an orthopaedic perspective, these findings emphasize that postoperative osteoporosis management should be integrated into the overall care pathway after fragility hip fracture surgery. Surgical fixation or arthroplasty can restore mechanical stability, but pharmacological secondary prevention remains necessary to reduce subsequent fracture risk and support skeletal recovery. The present findings suggest that medication selection should be linked to the dominant postoperative goal, such as early refracture prevention, longer-term bone recovery, or integrated rehabilitation planning.

The above findings should still be interpreted cautiously because of several limitations. First, the number of included studies was limited; some outcomes lacked direct comparisons, refracture events were few, and several comparisons involved zero events. Under these conditions, some refracture estimates were extreme and sensitive to prior specification, with wide CrIs and potentially unstable treatment rankings. Second, studies differed in baseline characteristics, fracture type, surgical procedures, treatment-initiation timing, follow-up duration, and outcome measurement, which may have affected comparability and transitivity. Treatment timing relative to fracture or surgery may influence bone loss, skeletal recovery, and rehabilitation, and could partly contribute to differences between antiresorptive and anabolic strategies. However, exact initiation timing was inconsistently reported and often intertwined with medication class, fracture type, and follow-up duration. Given the small number of studies and incomplete reporting, reliable subgroup analyses or network meta-regression according to treatment timing, surgical procedure, or fracture type were not feasible. The synthesis also combined RCTs and non-randomized studies; all 11 non-randomized studies had a high overall risk of bias, and the pooled estimates may therefore have been affected by residual confounding and selection bias. Although design-specific analyses would have been preferable, connected RCT networks were unavailable for most outcomes. Third, functional and pain outcomes, including HHS and VAS, reflect postoperative recovery and may be influenced by surgical and perioperative management, rehabilitation, analgesia, baseline functional and pain status, frailty, cognitive status, comorbidities, fracture characteristics, fixation quality, and postoperative complications. Because these factors were not consistently measured or controlled across studies, their individual contributions could not be quantified, limiting medication-specific interpretation; observed differences cannot be confidently attributed primarily to anti-osteoporosis medication choice. In addition, this review focused on efficacy outcomes and did not systematically assess adverse events, treatment discontinuation, adherence, renal considerations, hypocalcemia, or other clinically relevant safety outcomes. Therefore, the overall benefit–risk profiles and suitability of the medications could not be established. Fourth, sensitivity analyses showed that individual studies may have influenced some results, indicating that the robustness of related outcomes remains limited. GRADE assessments indicated limited certainty of evidence across outcomes, with detailed ratings presented in **Supplementary Tables 35**–**41**. Accordingly, the findings should be interpreted within the contemporary evidence window defined by this review, and the medication rankings should be regarded as trend-based references rather than a definitive clinical treatment hierarchy.

Based on these findings and their uncertainty, future research should be improved in several aspects. First, more high-quality randomized controlled trials targeting patients after surgery for fragility hip fractures are needed to improve the reliability of the evidence. Second, direct head-to-head comparisons between different anti-osteoporosis medications should be increased, especially among commonly used clinical medications such as ZOL, DEN, ALN, and TPTD, to reduce uncertainty from indirect comparisons. Third, future studies should standardise outcome measurement methods and follow-up time points for refracture, BMD, functional recovery, and pain as much as possible, and should strengthen prospective data collection, sample size calculation, reporting of loss to follow-up, and blinding of outcome assessment to improve methodological quality and comparability across studies. Finally, further attention should be paid to the timing of medication initiation, sequential therapy, safety, and medication adherence, so as to more accurately determine the applicable populations and clinical value of different medications in postoperative secondary fracture prevention.

## Conclusions

Following surgery for a fragility hip fracture, the available evidence suggested outcome- and time-specific efficacy patterns rather than consistent superiority of any anti-osteoporosis medication. ZOL, DEN, and ALN showed relatively favorable signals in some early refracture analyses, whereas TPTD showed a more favorable pattern for longer-term restoration of bone mass. These findings may provide context for goal-oriented clinical discussions but should not be used as the basis for treatment recommendations, given the limited and predominantly low-certainty evidence, few refracture events, and high risk of bias among non-randomized studies.

## Data Availability

The original contributions presented in the study are included in the article/**Supplementary Material**. Further inquiries can be directed to the corresponding authors.

## Supplementary material

Supplementary Material 1 Search strategies, methodological quality assessments, additional network meta-analysis results, sensitivity analyses, and GRADE evidence profiles.

Supplementary Material 2 Raw outcome data and analysis-ready datasets for the network meta-analysis.

Supplementary Material 3 MCMC trace and posterior density plots for the seven outcome-specific Bayesian models.
