# Supplementary material for: Effects of different anti-osteoporosis medications on re-fracture risk, bone mineral density, and functional recovery following hip fragility fracture surgery: a systematic review and network meta-analysis
Source: Front Endocrinol (Lausanne). 2026 Oct 20;17:1949781. doi: 10.3389/fendo.2026.1949781 (PMC13727487; doi:10.3389/fendo.2026.1949781)
Supplement: Supplementary file 1 [file Supplementaryfile1.docx]

***Supplementary Material 1***

**Supplementary Table** 1. Database-specific search strategies.

| **Database** | **Search strategy** |
| --- | --- |
| **PubMed** | ("Hip Fractures"[Mesh] OR "hip fracture*" OR "femoral neck fracture*" OR "intertrochanteric fracture*" OR "pertrochanteric fracture*" OR "trochanteric fracture*" OR "proximal femoral fracture*") AND ("Osteoporosis"[Mesh] OR osteoporo* OR "fragility fracture*" OR "osteoporotic fracture*") AND ("Zoledronic Acid"[Mesh] OR "zoledronic acid" OR zoledronate OR "Denosumab"[Mesh] OR denosumab OR "Teriparatide"[Mesh] OR teriparatide OR "Alendronate"[Mesh] OR alendronate OR "Risedronic Acid"[Mesh] OR risedronate OR bisphosphonate* OR "anti-osteoporosis treatment" OR "anti-osteoporotic medication*") AND (refracture OR "re-fracture" OR "secondary fracture" OR "subsequent fracture" OR "bone mineral density" OR BMD OR "Harris Hip Score" OR HHS OR "visual analog scale" OR "visual analogue scale" OR VAS OR pain OR function*) |
| **Embase** | ('hip fracture'/exp OR 'hip fracture*':ti,ab OR 'femoral neck fracture*':ti,ab OR 'intertrochanteric fracture*':ti,ab OR 'pertrochanteric fracture*':ti,ab OR 'trochanteric fracture*':ti,ab OR 'proximal femoral fracture*':ti,ab) AND ('osteoporosis'/exp OR osteoporo*:ti,ab OR 'fragility fracture*':ti,ab OR 'osteoporotic fracture*':ti,ab) AND ('zoledronic acid'/exp OR 'zoledronic acid':ti,ab OR zoledronate:ti,ab OR 'denosumab'/exp OR denosumab:ti,ab OR 'teriparatide'/exp OR teriparatide:ti,ab OR 'alendronate'/exp OR alendronate:ti,ab OR 'risedronate'/exp OR risedronate:ti,ab OR bisphosphonate*:ti,ab OR 'anti-osteoporosis treatment':ti,ab OR 'anti-osteoporotic medication*':ti,ab) AND (refracture:ti,ab OR 're-fracture':ti,ab OR 'secondary fracture':ti,ab OR 'subsequent fracture':ti,ab OR 'bone mineral density':ti,ab OR BMD:ti,ab OR 'Harris Hip Score':ti,ab OR HHS:ti,ab OR 'visual analog scale':ti,ab OR 'visual analogue scale':ti,ab OR VAS:ti,ab OR pain:ti,ab OR function*:ti,ab) |
| **Web of Science** | TS=("hip fracture*" OR "femoral neck fracture*" OR "intertrochanteric fracture*" OR "pertrochanteric fracture*" OR "trochanteric fracture*" OR "proximal femoral fracture*") AND TS=(osteoporo* OR "fragility fracture*" OR "osteoporotic fracture*") AND TS=("zoledronic acid" OR zoledronate OR denosumab OR teriparatide OR alendronate OR risedronate OR bisphosphonate* OR "anti-osteoporosis treatment" OR "anti-osteoporotic medication*") AND TS=(refracture OR "re-fracture" OR "secondary fracture" OR "subsequent fracture" OR "bone mineral density" OR BMD OR "Harris Hip Score" OR HHS OR "visual analog scale" OR "visual analogue scale" OR VAS OR pain OR function*) |
| **Cochrane Library** | ("hip fracture*" OR "femoral neck fracture*" OR "intertrochanteric fracture*" OR "pertrochanteric fracture*" OR "trochanteric fracture*" OR "proximal femoral fracture*") AND (osteoporo* OR "fragility fracture*" OR "osteoporotic fracture*") AND ("zoledronic acid" OR zoledronate OR denosumab OR teriparatide OR alendronate OR risedronate OR bisphosphonate* OR "anti-osteoporosis treatment" OR "anti-osteoporotic medication*") AND (refracture OR "re-fracture" OR "secondary fracture" OR "subsequent fracture" OR "bone mineral density" OR BMD OR "Harris Hip Score" OR HHS OR "visual analog scale" OR "visual analogue scale" OR VAS OR pain OR function*) |

**Supplementary Table** 2. Methodological Index for Non-Randomized Studies (MINORS) scoring criteria.

| **MINORS item** | **Score 0** | **Score 1** | **Score 2** |
| --- | --- | --- | --- |
| A. A clearly stated aim | NR | PR | WE |
| B. Inclusion of consecutive patients | NR | PR | WE |
| C. Prospective collection of data | NR | PR | WE |
| D. Endpoints appropriate to the aim of the study | NR | PR | WE |
| E. Unbiased assessment of the study endpoint | NR | PR | WE |
| F. Follow-up period appropriate to the aim of the study | NR | PR | WE |
| G. Loss to follow-up less than 5% | NR | PR | WE |
| H. Prospective calculation of the study size | NR | PR | WE |
| I. An adequate control group | NR | PR | WE |
| J. Contemporary groups | NR | PR | WE |
| K. Baseline equivalence of groups | NR | PR | WE |
| L. Adequate statistical analyses | NR | PR | WE |

**NR: not reported (0 point); PR: partially reported (1 point); WE: well established (2 points).**

A: the question addressed should be precise and relevant in the light of available literature.

B: all patients potentially fit for inclusion (satisfying the criteria for inclusion) have been included in the study during the study period; exclusions and reasons for exclusion should be described.

C: data were collected according to a protocol established before the beginning of the study.

D: unambiguous explanation of the criteria used to evaluate the main outcome, which should be in accordance with the question addressed by the study; endpoints should be assessed on an intention-to-treat basis when applicable.

E: blind evaluation of objective endpoints and double-blind evaluation of subjective endpoints should be used; otherwise, the reasons for not blinding should be stated.

F: the follow-up should be sufficiently long to allow assessment of the main endpoint and possible adverse events.

G: all patients should be included in the follow-up; otherwise, the proportion lost to follow-up should not exceed the proportion experiencing the major endpoint.

H: information should be provided on the size of detectable difference of interest, confidence interval, statistical significance level, and power estimates when comparing outcomes.

I: the control group should be appropriate in relation to the study question and should allow a clinically meaningful comparison.

J: the groups should be managed during the same period to avoid time-related bias.

K: the baseline characteristics of the groups should be comparable, or differences should be appropriately reported and considered.

L: statistical analyses should be appropriate for the study design, data type, and comparison between groups.

**Supplementary Table** 3. Clinical characteristics, potential effect modifiers, and BMD assessment details of included studies relevant to assessment of transitivity.

| **Study** | **Fracture type** | **Baseline BMD / osteoporosis severity** | **Treatment initiation** | **Follow-up duration** | **Calcium / vitamin D supplementation** |
| --- | --- | --- | --- | --- | --- |
| Li Y et al. (2016) | Osteoporotic intertrochanteric fracture | DXA-confirmed osteoporosis; hip BMD: ZOL 0.65, CTRL 0.69 g/cm² | ZOL on postoperative day 3; 12-month regimen | 12 months | Both: Caltrate D 600 mg/day + calcitriol 0.25 mg/day |
| Liu Z et al. (2019) | Osteoporotic intertrochanteric fracture | DXA osteoporosis; premedication BMD 0.70±0.06 vs 0.69±0.05; site NA; T-score ≤−2.5 or ≤−1 with prior fragility fracture | ZOL within 1 week after fracture; exact postoperative day NA | 24 months | Both: calcium carbonate 600 mg/day + active VitD3 400 IU/day |
| Koutalos AA et al. (2022) | Low-energy hip fracture: femoral neck or pertrochanteric | Vitamin D measured; baseline BMD/osteoporosis severity NA | ZOL and high-dose VitD on postoperative day 2 during hospitalization | 12 months | Both: daily Ca/VitD; active group additionally VitD 25,000 IU/week for 2 months and Ca 1,000 mg/day |
| Kim SJ et al. (2022) | Osteoporotic hip fracture: intertrochanteric or femoral neck | Hip T-score: DEN −2.5±0.6, ZOL −2.5±0.7 | After surgery; exact first-dose timing NA | Mean 2.5 years (range 2.0–7.2) | NA; vitamin D level measured |
| Huang TW et al. (2016), intertrochanteric | Osteoporotic intertrochanteric fracture (AO/OTA 31-A1) | Osteoporotic population; exact group BMD NA | TPTD initiated on the day of surgery; postoperative treatment | 12 months | All groups received Ca/VitD; dose NA |
| Kim SJ et al. (2018) | Unstable intertrochanteric fracture (AO/OTA A2/A3) | Osteoporosis (T-score ≤−2.5); group T-scores approximately −3.2 and −3.4 | TPTD from the day of surgery for 8 weeks | Mean 1.9 years (0.8–3.0); HHS/VAS at 6 months | NA |
| Kim SJ et al. (2019) | Unstable intertrochanteric fracture (AO/OTA 31-A2/A3) | Osteoporosis; group T-scores approximately −3.2 and −3.4 | Daily TPTD postoperatively for 2 months; exact first day NA | Mean 1.6 years (0.8–3.0); HHS/VAS at 6 months/final follow-up | NA |
| Hosseini Khameneh M et al. (2023) | Pertrochanteric fracture | Baseline BMD/osteoporosis severity NA | TPTD postoperatively for 3 months; exact first dose NA | 12 months | CTRL: Ca 1,000 mg/day + VitD 800 IU/day; TPTD group additionally received TPTD |
| Tanavalee C et al. (2025) | Pertrochanteric fracture (AO/OTA 31-A2/A3) | Total hip BMD 0.631±0.211 vs 0.598±0.220; femoral-neck BMD 0.518±0.156 vs 0.517±0.201 g/cm² | Daily TPTD from 48 h (postoperative day 2) for 12 weeks | 24 weeks (6 months) | All: calcium carbonate 1,000 mg/day + VitD2 20,000 IU/week |
| Lee SY et al. (2023) | Intertrochanteric or femoral neck fracture | Osteoporosis (T-score <−2.5); femoral-neck BMD measured 5 days post-op: approximately 0.481 vs 0.471 g/cm² | Weekly TPTD for ≥3 months; exact first-dose timing NA | 12 months | All received Ca/VitD supplements; dose NA |
| Ooi CS et al. (2024) | Low-energy proximal femoral fracture | Osteoporosis; femur T-score at 3 months −3.0±0.6 vs −3.1±0.9; baseline BMD NA | Postoperative TPTD/DEN; exact first-dose timing NA | 24 months | NA |
| Lu X et al. (2025) | Osteoporotic intertrochanteric fracture (stable/unstable) | Baseline T-score: DEN −2.91±0.34, CTRL −2.87±0.37 | DEN postoperatively every 6 months; exact first-dose timing NA | 12 months | All: calcium carbonate D3 + calcitriol; dose NA |
| Wang Y et al. (2025) | Osteoporotic low-energy femoral neck fracture | Osteoporotic population; BMD NA | DEN within 1 week following surgery; every 6 months | 3 years | NA |
| Wang KM et al. (2022) | Osteoporotic femoral intertrochanteric fracture | Femoral-neck T-score ≤−2.5; group values NA | Postoperative ALN 70 mg weekly; exact first dose NA | 12 months | NA |
| Huang TW et al. (2016), femoral neck | Osteoporotic femoral neck fracture | Osteoporosis; contralateral hip T-score: TPTD −4.2±1.0, CTRL −4.3±1.2 | Postoperative TPTD for ≥12 months; exact first dose NA | Mean 26.2–27.1 months; clinical outcomes through 12 months | Both: Ca 600 mg/day + VitD3 800 IU/day |
| Rana A et al. (2021) | Osteoporotic intertrochanteric fracture | DXA T-score <−2.5 at lumbar spine/opposite hip; hip BMD approximately 0.537 vs 0.561 | TPTD from the first postoperative day | 6 months | Both received standard Ca/VitD; dose NA |
| Aggarwal HO et al. (2025) | Osteoporotic intertrochanteric fracture | T-score: TPTD −3.04±0.43, CTRL −3.17±0.73; hip BMD assessed | Postoperative TPTD 20 μg/day for 24 weeks; exact first day NA | 24 weeks (6 months) | CTRL received calcium; dose and VitD use NA |
| Malouf-Sierra J et al. (2017) | Recent pertrochanteric hip fracture (AO/OTA 31-A1/A2) | Low bone mass (T-score ≤−2.0); baseline BMD 0.602–0.862 g/cm², site-dependent | Median 15 days from fracture to first dose; within 2 weeks after fixation | 78 weeks (approximately 18 months) | Both: calcium 800 mg/day + VitD 800 IU/day |

BMD, bone mineral density; DXA, dual-energy X-ray absorptiometry; ZOL, zoledronic acid; CTRL, Background therapy control (Ca/VitD ± placebo); Ca, calcium; VitD, vitamin D; IU, international units; TPTD, teriparatide; DEN, denosumab; ALN, alendronate; RIS, risedronate; HHS, Harris Hip Score; VAS, visual analogue scale; y, years; m, months; wk, weeks; NA, not available/not reported. Values are presented as reported; where group-specific values are shown, the order follows the source study. Baseline patient characteristics and surgical procedures are presented in Table 1.

**Panel B.** Hip BMD measurement characteristics of studies contributing to BMD outcomes.

| **Study** | **BMD format used in NMA** | **Hip site(s) reported** | **Measurement method/device** | **BMD follow-up time point** |
| --- | --- | --- | --- | --- |
| Li Y et al. (2016) | g/cm² | Uninjured-side/healthy-side hip: femoral neck, Ward's triangle, and greater trochanter; existing S3 values correspond to the femoral neck. | DXA; device not available | 12 months |
| Rana A et al. (2021) | g/cm² | Contralateral/opposite hip; exact subregion not available | DXA; device not available | 6 months |
| Malouf-Sierra J et al. (2017) | g/cm² | Contralateral unfractured hip: femoral neck and total hip | DXA using Hologic or Lunar equipment; cross-calibrated and centrally assessed | 26, 52, and 78 weeks; 78-week value extracted |
| Wang KM et al. (2022) | T-score | Femoral neck; laterality not available | DXA; device not available | 12 months |
| Ooi CS et al. (2024) | T-score | Femoral neck opposite the surgical site | DXA; Explorer Hologic | 3 and 24 months; 24-month value extracted |
| Aggarwal HO et al. (2025) | g/cm² and T-score | Hip reported; exact subregion and laterality not available | Method/device not available | 24 weeks |
| Lu X et al. (2025) | T-score | Contralateral uninjured proximal femur | DXA; device not available | Baseline and 12 months; 12-month value extracted |

Note. NA, not available. BMD expressed in g/cm² and T-scores were analyzed as separate outcomes. The table reports the hip site(s), imaging method/device, and follow-up time point(s) reported in BMD-contributing studies. When multiple sites or time points were reported, they are listed as reported; the endpoint entered into the network followed the data-extraction record. Because hip subregions and reporting formats were not sufficiently uniform across studies, a single site-specific analysis was not feasible.

**Supplementary Table** 4. Bayesian model specifications and numerical convergence diagnostics.

| **Outcome** | **Likelihood** | **Link function** | **Model** | **Chains** | **Adaptation iterations** | **Simulation iterations** | **Maximum PSRF (Upper C.I.)** | **Minimum ESS** |
| --- | --- | --- | --- | --- | --- | --- | --- | --- |
| Refracture rate (12 months) | Binomial | Logit | Random-effects consistency model | 4 | 50,000 | 100,000 | 1.065 | 177.75 |
| Hip refracture rate (12 months) | Binomial | Logit | Random-effects consistency model | 4 | 50,000 | 100,000 | 1.009 | 295.17 |
| Refracture rate (24 months) | Binomial | Logit | Random-effects consistency model | 4 | 5,000 | 20,000 | 1.002 | 5848.20 |
| HHS | Normal | Identity | Random-effects consistency model | 4 | 20,000 | 50,000 | 1.000 | 41405.05 |
| VAS | Normal | Identity | Random-effects consistency model | 4 | 20,000 | 50,000 | 1.000 | 29502.86 |
| BMD (g/cm²) | Normal | Identity | Random-effects consistency model | 4 | 20,000 | 50,000 | 1.000 | 11233.76 |
| BMD (T-score) | Normal | Identity | Random-effects consistency model | 4 | 20,000 | 50,000 | 1.000 | 23764.93 |

PSRF, potential scale reduction factor; ESS, effective sample size; HHS, Harris Hip Score; VAS, visual analogue scale; BMD, bone mineral density. Bayesian network meta-analyses were performed using random-effects consistency models with four Markov chains. Model convergence was assessed using PSRF and ESS.

**Supplementary Table** 5. Risk of Bias in Non-randomized Studies of Interventions (ROBINS-I) assessment.

| **Study** | **D1** | **D2** | **D3** | **D4** | **D5** | **D6** | **D7** | **Overall** |
| --- | --- | --- | --- | --- | --- | --- | --- | --- |
| Liu Z, et al. (2019) | Low | Intermediate | Intermediate | High | Low | High | Intermediate | High |
| Koutalos AA, et al. (2022) | Low | Intermediate | Intermediate | High | Low | High | Intermediate | High |
| Kim SJ, et al. (2022) | Low | Intermediate | Intermediate | High | Low | High | Intermediate | High |
| Huang TW, et al. (2016) | Low | High | Intermediate | High | Intermediate | High | Intermediate | High |
| Kim SJ, et al. (2018) | Low | High | Intermediate | High | Intermediate | High | Intermediate | High |
| Kim SJ, et al. (2019) | Low | High | Intermediate | High | Intermediate | High | Intermediate | High |
| Ooi CS, et al. (2024) | Low | High | Intermediate | High | Low | High | Intermediate | High |
| Lu X, et al. (2025) | Low | High | Intermediate | High | Low | High | Intermediate | High |
| Wang Y, et al. (2025) | Low | High | Intermediate | High | Low | High | Intermediate | High |
| Wang KM, et al. (2022) | Low | Intermediate | Intermediate | High | Low | High | Intermediate | High |
| Huang TW, et al. (2016) | Low | Intermediate | Intermediate | High | Low | High | Intermediate | High |

D1, bias due to confounding; D2, bias due to selection of participants; D3, bias in classification of interventions; D4, bias due to deviations from intended interventions; D5, bias due to missing data; D6, bias in measurement of outcomes; D7, bias in selection of the reported results.

**Supplementary Table** 6. Heterogeneity analysis of 12-month hip refracture rate.

| **t1** | **t2** | **I^2^.pair(%)** | **I^2^.cons(%)** | **Incons.p** |
| --- | --- | --- | --- | --- |
| CTRL | ZOL | NA | NA | NA |
| CTRL | TPTD | 0.00 | 0.00 | NA |
| CTRL | DEN | 0.00 | 0.00 | NA |
| CTRL | ALN+TPTD | NA | NA | NA |
| TPTD | ALN+TPTD | NA | NA | NA |

CTRL = Background therapy control (Ca/VitD ± placebo); ZOL = Zoledronic acid; DEN = Denosumab; TPTD = Teriparatide; RIS = Risedronate; ALN+TPTD = pre-fracture alendronate exposure followed by postoperative teriparatide treatment; NA = Not Applicable.

**Supplementary Table** 7. Ranking probabilities for 12-month hip refracture rate.

| **Intervention** | **Rank 1** | **Rank 2** | **Rank 3** | **Rank 4** | **Rank 5** |
| --- | --- | --- | --- | --- | --- |
| CTRL | 0.00 | 0.00 | 0.06 | 0.33 | 0.60 |
| ZOL | 0.93 | 0.04 | 0.02 | 0.01 | 0.01 |
| TPTD | 0.00 | 0.04 | 0.18 | 0.47 | 0.31 |
| DEN | 0.04 | 0.57 | 0.32 | 0.06 | 0.01 |
| ALN+TPTD | 0.03 | 0.36 | 0.42 | 0.13 | 0.07 |

CTRL = Background therapy control (Ca/VitD ± placebo); ZOL = Zoledronic acid; DEN = Denosumab; TPTD = Teriparatide; RIS = Risedronate; ALN+TPTD = pre-fracture alendronate exposure followed by postoperative teriparatide treatment.

**Supplementary Table** 8. Leave-one-out sensitivity analysis of 12-month hip refracture rate.

| **Removed study** | **Key random-effects result** | **Rank-probability ranking** | **Interpretation** |
| --- | --- | --- | --- |
| Huang TW 2016a | ALN+TPTD vs CTRL: -1.87 (-5.77, 1.11); DEN vs CTRL: -2.32 (-4.85, -0.24); TPTD vs CTRL: -0.68 (-3.70, 2.07) | ZOL > CTRL > DEN > TPTD > ALN+TPTD | Results remained stable after excluding this study, with no substantial change in treatment ranking. |
| Huang TW 2016b | DEN vs CTRL: -2.35 (-4.91, -0.20); TPTD vs CTRL: 0.22 (-4.04, 4.51); ZOL vs CTRL: -21.26 (-66.66, -1.49) | ZOL > DEN > CTRL > TPTD | ALN+TPTD node was removed after excluding this study; remaining treatment effects were consistent. |
| Li Y 2016 | ALN+TPTD vs CTRL: -1.78 (-5.74, 1.06); DEN vs CTRL: -2.35 (-4.83, -0.35); TPTD vs CTRL: -0.38 (-2.58, 1.74) | CTRL > DEN > TPTD > ALN+TPTD | Results remained stable after excluding this study, with no substantial change in treatment ranking. |
| Lu X 2025 | ALN+TPTD vs CTRL: -1.77 (-5.73, 1.27); DEN vs CTRL: -2.81 (-6.81, 0.20); TPTD vs CTRL: -0.40 (-2.76, 1.91) | ZOL > DEN > CTRL > ALN+TPTD > TPTD | Results remained stable after excluding this study, with no substantial change in treatment ranking. |
| Wang Y 2025 | ALN+TPTD vs CTRL: -1.74 (-5.44, 0.97); DEN vs CTRL: -2.06 (-5.82, 0.72); TPTD vs CTRL: -0.41 (-2.52, 1.63) | ZOL > CTRL > DEN > TPTD > ALN+TPTD | Results remained stable after excluding this study, with no substantial change in treatment ranking. |

CTRL = Background therapy control (Ca/VitD ± placebo); ZOL = Zoledronic acid; DEN = Denosumab; TPTD = Teriparatide; RIS = Risedronate; ALN = Alendronate; ALN+TPTD = pre-fracture alendronate exposure followed by postoperative teriparatide treatment;NA = Not Applicable.

**Supplementary Table** 9. Leave-one-out sensitivity analysis of 12-month overall refracture rate.

| **Removed study** | **Key random-effects result** | **Rank-probability ranking** | **Interpretation** |
| --- | --- | --- | --- |
| Huang TW 2016a | ALN vs CTRL: 21.93 (1.87, 57.33); ALN+TPTD vs CTRL: 0.89 (-1.90, 3.68); DEN vs CTRL: 2.63 (0.41, 5.37); TPTD vs CTRL: 0.61 (-2.15, 3.42) | ZOL > ALN > DEN > ALN+TPTD > TPTD > CTRL | Results remained stable after excluding this study, with no substantial change in treatment ranking. |
| Huang TW 2016b | ALN vs CTRL: 16.93 (1.86, 75.53); DEN vs CTRL: 2.61 (0.40, 5.31); TPTD vs CTRL: 0.11 (-2.75, 2.99); ZOL vs CTRL: 28.79 (3.03, 71.79) | ZOL > ALN > DEN > TPTD > CTRL | ALN+TPTD node disappeared after exclusion; overall treatment ranking remained stable. |
| Li Y 2016 | ALN vs CTRL: 17.97 (2.61, 50.32); ALN+TPTD vs CTRL: 0.82 (-1.31, 2.87); DEN vs CTRL: 2.56 (0.69, 4.97); TPTD vs CTRL: 0.41 (-1.25, 1.98) | ALN > DEN > ALN+TPTD > TPTD > CTRL | Results remained stable after excluding this study, with no substantial change in treatment ranking. |
| Lu X 2025 | ALN vs CTRL: 24.69 (3.70, 51.65); ALN+TPTD vs CTRL: 0.80 (-1.62, 3.17); DEN vs CTRL: 2.76 (-0.21, 6.78); TPTD vs CTRL: 0.42 (-1.46, 2.20) | ALN > ZOL > DEN > ALN+TPTD > TPTD > CTRL | Results remained stable after excluding this study, with no substantial change in treatment ranking. |
| Wang KM 2022 | ALN+TPTD vs CTRL: 0.81 (-1.32, 2.89); DEN vs CTRL: 2.59 (0.67, 5.02); TPTD vs CTRL: 0.41 (-1.26, 2.03); ZOL vs CTRL: 23.97 (2.30, 74.67) | ZOL > DEN > ALN+TPTD > TPTD > CTRL | ALN node disappeared after exclusion because this study provided the only ALN comparison; remaining treatment effects were consistent. |
| Wang Y 2025 | ALN vs CTRL: 15.14 (1.99, 41.77); ALN+TPTD vs CTRL: 0.80 (-1.63, 3.14); DEN vs CTRL: 2.60 (-0.40, 6.62); TPTD vs CTRL: 0.39 (-1.44, 2.19) | ZOL > ALN > DEN > ALN+TPTD > TPTD > CTRL | Results remained stable after excluding this study, with no substantial change in treatment ranking. |

CTRL = Background therapy control (Ca/VitD ± placebo); ZOL = Zoledronic acid; DEN = Denosumab; TPTD = Teriparatide; RIS = Risedronate; ALN = Alendronate; ALN+TPTD = pre-fracture alendronate exposure followed by postoperative teriparatide treatment;NA = Not Applicable.

**Supplementary Table** 10. Heterogeneity analysis of 12-month overall refracture rate.

| **t1** | **t2** | **I^2^.pair(%)** | **I^2^.cons(%)** | **Incons.p** |
| --- | --- | --- | --- | --- |
| CTRL | ZOL | NA | NA | NA |
| CTRL | TPTD | 0.00 | 0.00 | NA |
| CTRL | DEN | 0.00 | 0.00 | NA |
| CTRL | ALN | NA | NA | NA |
| CTRL | ALN+TPTD | NA | NA | NA |
| TPTD | ALN+TPTD | NA | NA | NA |

CTRL = Background therapy control (Ca/VitD ± placebo); ZOL = Zoledronic acid; DEN = Denosumab; TPTD = Teriparatide; RIS = Risedronate; ALN = Alendronate; ALN+TPTD = pre-fracture alendronate exposure followed by postoperative teriparatide treatment; NA = Not Applicable.

**Supplementary Table** 11. Heterogeneity analysis of 24-month overall refracture rate.

| **t1** | **t2** | **I^2^.pair(%)** | **I^2^.cons(%)** | **Incons.p** |
| --- | --- | --- | --- | --- |
| CTRL | ZOL | NA | 49.01 | 0.44 |
| CTRL | DEN | NA | 39.05 | 0.34 |
| ZOL | DEN | NA | 47.09 | 0.30 |
| DEN | TPTD | NA | NA | NA |

CTRL = Background therapy control (Ca/VitD ± placebo); ZOL = Zoledronic acid; TPTD = Teriparatide; DEN = Denosumab. NA = Not Applicable.

**Supplementary Table** 12. Node-splitting analysis of 24-month overall refracture rate.

| **Comparison** | **Direct** | **Indirect** | **Network** | **P.value** |
| --- | --- | --- | --- | --- |
| ZOLvsCTRL | 0.69(0.06,8.30) | 0.08 (0.00, 3.60) | 0.43 (0.04, 3.10) | 0.27 |
| DENvsCTRL | 0.10(0.01, 1.60) | 0.88 (0.02, 39.) | 0.24 (0.02, 2.10) | 0.28 |
| DENvsZOL | 1.30(0.09, 21.) | 0.15 (0.00, 6.00) | 0.56 (0.06, 5.60) | 0.28 |

CTRL = Background therapy control (Ca/VitD ± placebo); DEN = Denosumab; ZOL = Zoledronic acid.

**Supplementary Table** 13. Ranking probabilities for 12-month overall refracture rate.

| **Intervention** | **Rank 1** | **Rank 2** | **Rank 3** | **Rank 4** | **Rank 5** | **Rank 6** |
| --- | --- | --- | --- | --- | --- | --- |
| CTRL | 0.00 | 0.00 | 0.00 | 0.08 | 0.24 | 0.68 |
| ZOL | 0.41 | 0.54 | 0.04 | 0.01 | 0.00 | 0.00 |
| TPTD | 0.00 | 0.00 | 0.02 | 0.25 | 0.52 | 0.20 |
| DEN | 0.00 | 0.07 | 0.84 | 0.07 | 0.02 | 0.00 |
| ALN | 0.59 | 0.38 | 0.02 | 0.00 | 0.00 | 0.00 |
| ALN+TPTD | 0.00 | 0.00 | 0.08 | 0.60 | 0.21 | 0.11 |

CTRL = Background therapy control (Ca/VitD ± placebo); ZOL = Zoledronic acid; DEN = Denosumab; TPTD = Teriparatide; RIS = Risedronate; ALN = Alendronate; ALN+TPTD = pre-fracture alendronate exposure followed by postoperative teriparatide treatment.

**Supplementary Table** 14. Ranking probabilities for 24-month overall refracture rate.

| **Intervention** | **Rank 1** | **Rank 2** | **Rank 3** | **Rank 4** |
| --- | --- | --- | --- | --- |
| CTRL | 0.01 | 0.04 | 0.16 | 0.79 |
| ZOL | 0.08 | 0.22 | 0.57 | 0.13 |
| DEN | 0.09 | 0.64 | 0.22 | 0.05 |
| TPTD | 0.82 | 0.10 | 0.05 | 0.03 |

CTRL = Background therapy control (Ca/VitD ± placebo); ZOL = Zoledronic acid; TPTD = Teriparatide; DEN = Denosumab.

**Supplementary Table** 15. Leave-one-out sensitivity analysis of 24-month overall refracture rate.

| **Removed study** | **Key random-effects result** | **Rank-probability ranking** | **Interpretation** |
| --- | --- | --- | --- |
| Kim SJ 2022 | DEN vs CTRL: 2.25 (-0.42, 5.21); TPTD vs CTRL: 3.79 (-0.12, 7.88); ZOL vs CTRL: 0.37 (-2.11, 2.82) | TPTD > DEN > ZOL > CTRL | Results remained stable after excluding this study, with no substantial change in treatment ranking. |
| Liu Z 2019 | DEN vs CTRL: 2.27 (-0.46, 5.28); TPTD vs CTRL: 3.76 (-0.16, 8.00); ZOL vs CTRL: 2.58 (-1.35, 6.62) | TPTD > ZOL > DEN > CTRL | Results remained stable after excluding this study, with no substantial change in treatment ranking. |
| Ooi CS 2024 | DEN vs CTRL: 1.43 (-0.76, 3.78); ZOL vs CTRL: 0.85 (-1.13, 3.17) | DEN > ZOL > CTRL | Results remained stable after excluding this study, with no substantial change in treatment ranking. |
| Wang Y 2025 | DEN vs CTRL: 0.07 (-2.62, 2.70); TPTD vs CTRL: 1.59 (-1.84, 5.11); ZOL vs CTRL: 0.37 (-1.25, 2.01) | TPTD > ZOL > CTRL > DEN | Results remained stable after excluding this study, with no substantial change in treatment ranking. |

CTRL = Background therapy control (Ca/VitD ± placebo); ZOL = Zoledronic acid; DEN = Denosumab; TPTD = Teriparatide; RIS = Risedronate; ALN = Alendronate; NA = Not Applicable.

**Supplementary Table** 16. Sensitivity of refracture estimates to prior specifications.

**A. 12-month hip refracture**

| **Comparison** | **Original vague prior** | **N(0, 10²) + U(0, 2)** | **N(0, 5²) + U(0, 1)** |
| --- | --- | --- | --- |
| TPTD vs CTRL | 0.67 (0.074, 5.58) | 0.681 (0.0758, 5.53) | 0.647 (0.111, 3.19) |
| ALN+TPTD vs CTRL | 0.17 (3.1e-3, 2.86) | 0.176 (0.00468, 2.86) | 0.187 (0.00891, 1.64) |
| ALN+TPTD vs TPTD | 0.26 (4.1e-3, 5.43) | 0.259 (0.00619, 5.19) | 0.289 (0.0119, 3.47) |
| ZOL vs CTRL | 1.0e-9 (2.3e-32, 0.29) | 6.18e-4 (9.69e-11, 0.957) | 0.0168 (6.71e-6, 1.37) |
| ZOL vs TPTD | 1.6e-9 (4.3e-32, 0.53) | 8.94e-4 (1.30e-10, 2.1) | 0.0257 (8.33e-6, 3.15) |
| ZOL vs ALN+TPTD | 6.8e-9 (2.0e-31, 3.25) | 0.00351 (4.70e-10, 14.2) | 0.0931 (2.56e-5, 19.4) |
| DEN vs CTRL | 0.093 (8.3e-3, 0.72) | 0.0977 (0.00875, 0.709) | 0.106 (0.0138, 0.5) |
| DEN vs TPTD | 0.14 (5.6e-3, 2.85) | 0.142 (0.00585, 2.74) | 0.161 (0.0129, 1.69) |
| DEN vs ALN+TPTD | 0.53 (0.013, 50.85) | 0.555 (0.0136, 33.6) | 0.557 (0.0299, 16.9) |
| DEN vs ZOL | 9.1e+7 (0.26, 3.5e+30) | 155 (0.0647, 1.01e+9) | 6.09 (0.0479, 1.77e+4) |

**B. 12-month overall refracture**

| **Comparison** | **Original vague prior** | **N(0, 10²) + U(0, 2)** | **N(0, 5²) + U(0, 1)** |
| --- | --- | --- | --- |
| TPTD vs CTRL | 0.67 (0.14, 3.50) | 0.666 (0.154, 3.08) | 0.661 (0.241, 1.87) |
| ALN+TPTD vs CTRL | 0.45 (0.056, 3.78) | 0.446 (0.0687, 3.16) | 0.447 (0.126, 1.61) |
| ALN+TPTD vs TPTD | 0.68 (0.078, 5.65) | 0.67 (0.0957, 4.76) | 0.675 (0.177, 2.57) |
| ZOL vs CTRL | 1.5e-10 (1.0e-39, 0.066) | 4.33e-4 (2.30e-10, 0.365) | 0.00954 (5.42e-6, 0.484) |
| ZOL vs TPTD | 2.4e-10 (1.5e-39, 0.11) | 6.27e-4 (3.20e-10, 0.648) | 0.0142 (7.82e-6, 0.836) |
| ZOL vs ALN+TPTD | 3.4e-10 (2.0e-39, 0.18) | 9.31e-4 (4.33e-10, 1.05) | 0.0208 (1.14e-5, 1.35) |
| DEN vs CTRL | 0.075 (6.5e-3, 0.51) | 0.0765 (0.00743, 0.474) | 0.0828 (0.0111, 0.373) |
| DEN vs TPTD | 0.11 (5.9e-3, 1.33) | 0.113 (0.00709, 1.17) | 0.122 (0.0133, 0.757) |
| DEN vs ALN+TPTD | 0.16 (6.3e-3, 2.73) | 0.171 (0.00838, 2.25) | 0.182 (0.0173, 1.31) |
| DEN vs ZOL | 4.8e+8 (0.85, 7.9e+37) | 171 (0.132, 3.87e+8) | 8.74 (0.0979, 1.65e+4) |
| ALN vs CTRL | 1.9e-11 (2.1e-28, 0.061) | 2.89e-4 (8.53e-11, 0.371) | 0.0111 (8.41e-6, 0.541) |
| ALN vs TPTD | 3.1e-11 (3.0e-28, 0.10) | 4.23e-4 (1.20e-10, 0.668) | 0.0163 (1.19e-5, 0.949) |
| ALN vs ALN+TPTD | 4.6e-11 (4.4e-28, 0.16) | 6.26e-4 (1.63e-10, 1.1) | 0.0241 (1.69e-5, 1.49) |
| ALN vs ZOL | 0.99 (4.1e-19, 4.0e+23) | 0.751 (3.53e-8, 7.81e+6) | 1.15 (3.53e-4, 4.80e+3) |
| ALN vs DEN | 2.7e-10 (2.9e-27, 0.99) | 0.00394 (1.09e-9, 7.83) | 0.136 (8.61e-5, 11.5) |

**C. 24-month overall refracture**

| **Comparison** | **Original vague prior** | **N(0, 10²) + U(0, 2)** | **N(0, 5²) + U(0, 1)** |
| --- | --- | --- | --- |
| ZOL vs CTRL | 2.38 (0.33, 22.57) | 2.35 (0.307, 24.6) | 2.17 (0.649, 8.8) |
| DEN vs CTRL | 4.10 (0.47, 43.20) | 4.14 (0.458, 44.4) | 3.96 (0.986, 17.8) |
| DEN vs ZOL | 1.71 (0.17, 17.60) | 1.75 (0.164, 15.7) | 1.82 (0.402, 7.61) |
| TPTD vs CTRL | 18.51 (0.51, 9.5e+2) | 18.3 (0.474, 878) | 16.7 (1.45, 253) |
| TPTD vs ZOL | 7.67 (0.19, 3.3e+2) | 7.65 (0.173, 321) | 7.59 (0.61, 110) |
| TPTD vs DEN | 4.44 (0.25, 1.1e+2) | 4.35 (0.236, 94.6) | 4.14 (0.566, 41.3) |

N, normal distribution; U, uniform distribution; OR, odds ratio; CrI, credible interval; CTRL, Background therapy control (Ca/VitD ± placebo); ZOL, zoledronic acid; DEN, denosumab; TPTD, teriparatide; ALN, alendronate;ALN+TPTD = pre-fracture alendronate exposure followed by postoperative teriparatide treatment.

**Supplementary Table** 17. Heterogeneity analysis of hip BMD (g/cm²).

| **t1** | **t2** | **I^2^.pair(%)** | **I^2^.cons(%)** | **Incons.p** |
| --- | --- | --- | --- | --- |
| CTRL | ZOL | NA | NA | NA |
| CTRL | TPTD | 0.00 | 0.00 | NA |
| TPTD | RIS | NA | NA | NA |

BMD (g/cm²) = Bone Mineral Density (grams per square centimeter); CTRL = Background therapy control (Ca/VitD ± placebo); ZOL = Zoledronic acid; TPTD = Teriparatide; RIS = Risedronate. NA = Not Applicable.

**Supplementary Table** 18. Heterogeneity analysis of hip BMD (T-score).

| **t1** | **t2** | **I^2^.pair(%)** | **I^2^.cons(%)** | **Incons.p** |
| --- | --- | --- | --- | --- |
| CTRL | TPTD | NA | 57.17 | 0.44 |
| CTRL | DEN | NA | 69.71 | 0.52 |
| CTRL | ALN | NA | NA | NA |
| TPTD | DEN | NA | 67.24 | 0.52 |

BMD (T-score) = Bone Mineral Density T-score; CTRL = Background therapy control (Ca/VitD ± placebo); TPTD = Teriparatide; DEN = Denosumab; ALN = Alendronate. NA = Not Applicable.

**Supplementary Table** 19. Node-splitting analysis of hip BMD (T-score).

| **Comparison** | **Direct** | **Indirect** | **Network** | **P.value** |
| --- | --- | --- | --- | --- |
| CTRLvsTPTD | 0.19 (-0.74, 1.1) | 0.73 (-0.53, 2.) | 0.40 (-0.37, 1.1) | 0.35 |
| CTRLvsDEN | 0.53 (-0.35, 1.4) | -0.011 (-1.3, 1.3) | 0.36 (-0.39, 1.1) | 0.35 |
| TPTDvsDEN | -0.20 (-1.1, 0.70) | 0.33 (-0.94, 1.6) | -0.033 (-0.77, 0.71) | 0.35 |

BMD (T-score)= Bone Mineral Density T-score; CTRL = Background therapy control (Ca/VitD ± placebo); TPTD = Teriparatide; DEN = Denosumab.

**Supplementary Table** 20. Ranking probabilities for hip BMD (g/cm²).

| **Intervention** | **Rank 1** | **Rank 2** | **Rank 3** | **Rank 4** |
| --- | --- | --- | --- | --- |
| CTRL | 0.01 | 0.23 | 0.31 | 0.46 |
| ZOL | 0.86 | 0.07 | 0.05 | 0.03 |
| TPTD | 0.05 | 0.47 | 0.38 | 0.10 |
| RIS | 0.08 | 0.24 | 0.27 | 0.42 |

BMD (g/cm²) = Bone Mineral Density (grams per square centimeter); CTRL = Background therapy control (Ca/VitD ± placebo); ZOL = Zoledronic acid; TPTD = Teriparatide; RIS = Risedronate.

**Supplementary Table** 21. Ranking probabilities for hip BMD (T-score).

| **Intervention** | **Rank 1** | **Rank 2** | **Rank 3** | **Rank 4** |
| --- | --- | --- | --- | --- |
| CTRL | 0.00 | 0.06 | 0.17 | 0.77 |
| TPTD | 0.16 | 0.43 | 0.32 | 0.09 |
| DEN | 0.14 | 0.37 | 0.39 | 0.10 |
| ALN | 0.69 | 0.14 | 0.12 | 0.04 |

BMD (T-score) = Bone Mineral Density T-score; CTRL = Background therapy control (Ca/VitD ± placebo); TPTD = Teriparatide; DEN = Denosumab; ALN = Alendronate.

**Supplementary Table** 22. Sensitivity of BMD results to the assumed baseline-to-follow-up correlation coefficient.

| **Outcome** | **Assumed r** | **Comparison** | **MD** | **95% CrI** |
| --- | --- | --- | --- | --- |
| BMD g/cm² | 0.25 | ZOL vs CTRL | 0.1003 | -0.0276 to 0.2276 |
|  | 0.25 | TPTD vs CTRL | 0.0180 | -0.0694 to 0.1047 |
|  | 0.25 | RIS vs CTRL | 0.0040 | -0.1407 to 0.1486 |
|  | 0.50 (primary) | ZOL vs CTRL | 0.1007 | -0.0199 to 0.2191 |
|  | 0.50 (primary) | TPTD vs CTRL | 0.0180 | -0.0652 to 0.0982 |
|  | 0.50 (primary) | RIS vs CTRL | 0.0040 | -0.1337 to 0.1399 |
|  | 0.75 | ZOL vs CTRL | 0.1004 | -0.0115 to 0.2111 |
|  | 0.75 | TPTD vs CTRL | 0.0179 | -0.0611 to 0.0954 |
|  | 0.75 | RIS vs CTRL | 0.0042 | -0.1294 to 0.1361 |
| BMD T-score | 0.25 | TPTD vs CTRL | 0.4228 | -0.3707 to 1.1359 |
|  | 0.25 | DEN vs CTRL | 0.3858 | -0.3676 to 1.0849 |
|  | 0.25 | ALN vs CTRL | 0.6995 | -0.1486 to 1.5439 |
|  | 0.50 (primary) | TPTD vs CTRL | 0.3898 | -0.3821 to 1.1138 |
|  | 0.50 (primary) | DEN vs CTRL | 0.3632 | -0.3900 to 1.0831 |
|  | 0.50 (primary) | ALN vs CTRL | 0.6998 | -0.1912 to 1.5930 |
|  | 0.75 | TPTD vs CTRL | 0.3873 | -0.3716 to 1.1261 |
|  | 0.75 | DEN vs CTRL | 0.3609 | -0.3899 to 1.0922 |
|  | 0.75 | ALN vs CTRL | 0.6997 | -0.1941 to 1.5879 |

Note. CTRL = Background therapy control (Ca/VitD ± placebo); ZOL = Zoledronic acid; DEN = Denosumab; TPTD = Teriparatide; RIS = Risedronate;MD = mean difference;

The primary analysis assumed r = 0.50; sensitivity analyses used r = 0.25 and r = 0.75. SDchange = √(SDbaseline² + SDfollow-up² − 2r×SDbaseline×SDfollow-up). The direction and interpretation of the BMD estimates were qualitatively consistent across the three assumptions.

**Supplementary Table** 23. Leave-one-out sensitivity analysis of hip BMD (g/cm²).

| **Removed study** | **Key random-effects result** | **Rank-probability ranking** | **Interpretation** |
| --- | --- | --- | --- |
| Aggarwal HO 2025 | ALN: -0.15 (-0.34, 0.04); DEN: -0.14 (-0.39, 0.11); TPTD: -0.22 (-0.59, 0.14) | TPTD > ALN > DEN > CTRL | Results remained consistent after excluding this study, with no substantial changes in treatment effects or ranking. |
| Lu X 2025 | ALN: -0.15 (-0.34, 0.04); DEN: -0.05 (-0.47, 0.38); TPTD: -0.13 (-0.46, 0.21) | ALN > TPTD > DEN > CTRL | Results remained consistent after excluding this study, with no substantial changes in treatment effects or ranking. |
| Ooi CS 2024 | ALN: -0.15 (-0.34, 0.04); DEN: -0.14 (-0.39, 0.11); TPTD: -0.14 (-0.47, 0.20) | TPTD > DEN > ALN > CTRL | Results remained consistent after excluding this study, with no substantial changes in treatment effects or ranking. |
| Wang KM 2022 | DEN: -0.12 (-0.32, 0.09); TPTD: -0.17 (-0.41, 0.06) | TPTD > DEN > CTRL | Results remained consistent after excluding this study, with no substantial changes in treatment effects or ranking. |

BMD (g/cm²) = Bone Mineral Density (grams per square centimeter); CTRL = Background therapy control (Ca/VitD ± placebo); TPTD = Teriparatide; DEN = Denosumab; ALN = Alendronate; ZOL = Zoledronic acid.

**Supplementary Table** 24. Leave-one-out sensitivity analysis of hip BMD (T-score).

| **Removed study** | **Key random-effects result** | **Rank-probability ranking** | **Interpretation** |
| --- | --- | --- | --- |
| Aggarwal HO 2025 | ALN: -0.70 (-1.58, 0.17); DEN: -0.53 (-1.43, 0.36); TPTD: -0.73 (-2.01, 0.53) | TPTD > ALN > DEN > CTRL | Results remained consistent after excluding this study, without substantial changes in treatment effects or ranking. |
| Lu X 2025 | ALN: -0.70 (-1.58, 0.18); DEN: 0.01 (-1.27, 1.27); TPTD: -0.19 (-1.10, 0.72) | ALN > TPTD > DEN > CTRL | Results remained consistent after excluding this study, without substantial changes in treatment effects or ranking. |
| Ooi CS 2024 | ALN: -0.70 (-1.59, 0.18); DEN: -0.53 (-1.41, 0.37); TPTD: -0.19 (-1.09, 0.73) | ALN > DEN > TPTD > CTRL | Results remained consistent after excluding this study, without substantial changes in treatment effects or ranking. |
| Wang KM 2022 | DEN: -0.37 (-0.95, 0.24); TPTD: -0.40 (-0.97, 0.23) | TPTD > DEN > CTRL | Results remained consistent after excluding this study, without substantial changes in treatment effects or ranking. |

BMD (T-score) = Bone Mineral Density T-score; CTRL = Background therapy control (Ca/VitD ± placebo); TPTD = Teriparatide; DEN = Denosumab; ALN = Alendronate.

**Supplementary Table** 25. Heterogeneity analysis of HHS.

| **t1** | **t2** | **I^2^.pair(%)** | **I^2^.cons(%)** | **Incons.p** |
| --- | --- | --- | --- | --- |
| CTRL | TPTD | 92.67 | 92.69 | NA |
| CTRL | DEN | NA | NA | NA |
| CTRL | ALN | NA | NA | NA |
| CTRL | ZOL | NA | NA | NA |

HHS = Harris Hip Score; CTRL = Background therapy control (Ca/VitD ± placebo); TPTD = Teriparatide; DEN = Denosumab; ALN = Alendronate; ZOL = Zoledronic acid. NA = Not Applicable.

**Supplementary Table** 26. Ranking probabilities for HHS.

| **Intervention** | **Rank 1** | **Rank 2** | **Rank 3** | **Rank 4** | **Rank 5** |
| --- | --- | --- | --- | --- | --- |
| CTRL | 0 | 0.03 | 0.2 | 0.48 | 0.28 |
| TPTD | 0.17 | 0.4 | 0.3 | 0.1 | 0.03 |
| DEN | 0.2 | 0.24 | 0.23 | 0.16 | 0.18 |
| ALN | 0.56 | 0.21 | 0.12 | 0.06 | 0.05 |
| ZOL | 0.08 | 0.12 | 0.15 | 0.19 | 0.46 |

HHS = Harris Hip Score; CTRL = Background therapy control (Ca/VitD ± placebo); TPTD = Teriparatide; DEN = Denosumab; ALN = Alendronate; ZOL = Zoledronic acid.

**Supplementary Table** 27. Leave-one-out sensitivity analysis of HHS.

| **Removed study** | **Key random-effects result** | **Rank-probability ranking** | **Interpretation** |
| --- | --- | --- | --- |
| Kim SJ et al. (2018) | No significant differences; ALN 7.87 (-5.68, 21.42); TPTD 4.66 (-2.64, 11.96). | ALN > TPTD > DEN > ZOL > CTRL | Conclusion unchanged; heterogeneity high. |
| Kim SJ et al. (2019) | No significant differences; ALN 7.87 (-6.00, 21.74); TPTD 3.00 (-4.46, 10.46). | ALN > TPTD > DEN > ZOL > CTRL | Conclusion unchanged; heterogeneity high. |
| Hosseini Khameneh et al. (2023) | No significant differences; ALN 7.87 (-2.16, 17.90); TPTD 2.69 (-3.21, 8.59). | ALN > DEN > TPTD > ZOL > CTRL | Heterogeneity decreased but remained notable. |
| Tanavalee C et al. (2025) | No significant differences; ALN 7.87 (-4.84, 20.58); TPTD 5.21 (-1.52, 11.95). | ALN > TPTD > DEN > ZOL > CTRL | Conclusion unchanged; heterogeneity high. |
| Lu X et al. (2025) | No significant differences; ALN 7.87 (-4.62, 20.36); TPTD 4.41 (-1.70, 10.52). | ALN > TPTD > ZOL > CTRL | DEN node removed; conclusion unchanged. |
| Wang KM et al. (2022) | No significant differences; TPTD 4.41 (-1.70, 10.52); DEN 3.37 (-9.16, 15.90). | TPTD > DEN > ZOL > CTRL | ALN node removed; ranking not comparable. |
| Huang TW et al. (2016) | ALN and TPTD became statistically significant vs CTRL: ALN 7.87 (1.32, 14.42); TPTD 7.02 (2.78, 11.26). | ALN > TPTD > DEN > ZOL > CTRL | Largest reduction in heterogeneity; likely influential study. |
| Li Y et al. (2016) | No significant differences; ALN 7.87 (-4.62, 20.36); TPTD 4.41 (-1.70, 10.52). | ALN > TPTD > DEN > CTRL | ZOL node removed; conclusion unchanged. |

HHS = Harris Hip Score; MD = mean difference; CI = confidence interval; CTRL = Background therapy control (Ca/VitD ± placebo); TPTD = teriparatide; DEN = denosumab; ALN = alendronate; ZOL = zoledronic acid. Higher HHS indicates better function. Rankings were based on rank probabilities recalculated after each leave-one-out analysis. > indicates a more favorable rank-probability ranking, not necessarily a statistically significant difference.

**Supplementary Table** 28. Comparison of treatment effects between the primary network meta-analysis and the RCT-only sensitivity analysis.

Outcome: Harris Hip Score (HHS)

| **Comparison** | **Primary NMA (RCT + NRS), MD (95% CrI)** | **RCT-only NMA, MD (95% CrI)** | **Interpretation** |
| --- | --- | --- | --- |
| TPTD vs CTRL | 0.26 (-6.97, 7.13) | 6.59 (-4.21, 15.53) | Effect direction remained positive; credible intervals overlapped and included the null effect |
| ZOL vs CTRL | 0.50 (-14.58, 15.52) | -0.51 (-13.67, 12.53) | Effect estimates were highly consistent with no evidence of a significant difference |
| TPTD vs ZOL | -0.23 (-17.05, 16.18) | 7.16 (-4.21, 15.53) | Point estimates varied after restricting to RCTs, but uncertainty intervals remained overlapping and conclusions were unchanged |

Outcome: Visual Analog Scale (VAS)

| **Comparison** | **Primary NMA (RCT + NRS), MD (95% CrI)** | **RCT-only NMA, MD (95% CrI)** | **Interpretation** |
| --- | --- | --- | --- |
| ZOL vs CTRL | -0.99 (-3.43, 1.10) | -1.30 (-3.45, 0.85) | Effect magnitude and direction were consistent between analyses |
| TPTD vs CTRL | 0.90 (-1.16, 2.94) | -1.08 (-2.72, 0.57) | Effect estimate changed after excluding NRS, but credible intervals overlapped and statistical significance was unchanged |
| TPTD vs ZOL | 1.88 (-0.99, 5.10) | 0.22 (-2.46, 2.88) | No significant difference was observed in either analysis |

**Supplementary Table** 29. Comparison of treatment ranking probabilities between the primary network meta-analysis and the RCT-only sensitivity analysis.

Outcome: Harris Hip Score (HHS)

| **Treatment** | **Primary NMA Rank 1 probability** | **RCT-only NMA Rank 1 probability** |
| --- | --- | --- |
| CTRL | 0.00 | 0.048 |
| TPTD | 0.17 | 0.149 |
| ZOL | 0.08 | 0.802 |
| DEN | 0.20 | Not available |
| ALN | 0.56 | Not available |

Note: RCT-only analysis included only CTRL, TPTD, and ZOL; therefore, DEN and ALN were not available for ranking

Outcome: Visual Analog Scale (VAS)

| **Treatment** | **Primary NMA Rank 1 probability** | **RCT-only NMA Rank 1 probability** |
| --- | --- | --- |
| CTRL | 0.06 | 0.064 |
| ZOL | 0.70 | 0.834 |
| TPTD | 0.03 | 0.102 |
| DEN | 0.21 | Not available |

MD, mean difference; CrI, credible interval; NMA, network meta-analysis; RCT, randomized controlled trial; NRS, non-randomized study; CTRL, background therapy control; TPTD, teriparatide; ZOL, zoledronic acid; DEN, denosumab; ALN, alendronate.

Note: RCT-only sensitivity analyses were conducted for outcomes with sufficient randomized evidence (HHS and VAS). Treatment effects were considered consistent when the direction of effect remained unchanged and credible intervals showed substantial overlap between the primary and RCT-only analyses.

**Supplementary Table** 30. Comparison of HHS and VAS outcomes between the primary final-follow-up analysis and the exact-12-month sensitivity analysis.

| **Outcome** | **Comparison** | **Final follow-up analysis, MD (95% CrI)** | **12-month restricted sensitivity analysis, MD (95% CrI)** | **Interpretation** |
| --- | --- | --- | --- | --- |
| HHS | ALN vs CTRL | -7.88 (-22.66, 7.00) | -7.86 (-22.24, 6.61) | Consistent |
|  | DEN vs CTRL | -3.36 (-18.12, 11.44) | -3.35 (-17.72, 11.17) | Consistent |
|  | TPTD vs CTRL | 0.26 (-6.97, 7.13) | 4.09 (-6.34, 14.32) | Consistent |
|  | ZOL vs CTRL | 0.50 (-14.58, 15.52) | 0.46 (-14.16, 15.19) | Consistent |
| VAS | ZOL vs CTRL | -0.99 (-3.43, 1.10) | -0.35 (-1.84, 0.83) | Consistent |
|  | DEN vs CTRL | 0.05 (-2.84, 2.95) | -0.61 (-2.27, 1.06) | Consistent |
|  | TPTD vs CTRL | 0.90 (-1.16, 2.94) | -1.08 (-2.69, 0.55) | Consistent |

Note. MD = mean difference; CrI = credible interval. Final follow-up analyses included the longest available follow-up reported in each study. The 12-month restricted sensitivity analyses included only studies reporting outcomes at 12 months. Consistent results indicate that restricting follow-up duration did not materially alter treatment effects.

**Supplementary Table** 31. Node-splitting analysis of VAS.

| **Comparison** | **Direct** | **Indirect** | **Network** | **P.value** |
| --- | --- | --- | --- | --- |
| CTRLvsTPTD | -0.71(-3.2,1.80) | -2.00 (-8.00, 4.10) | -0.91 (-2.90, 1.20) | 0.62 |
| CTRLvsDEN | -0.62 (-4.90, 3.70) | 0.69 (-4.30, 5.60) | -0.05(-2.90, 2.80) | 0.61 |
| TPTDvsDEN | 1.40 (-2.90, 5.60) | 0.11 (-4.80, 5.00) | 0.85 (-2.00, 3.70) | 0.61 |

VAS = Visual Analog Scale score; CTRL = Background therapy control (Ca/VitD ± placebo); TPTD = Teriparatide; DEN = Denosumab.

**Supplementary Table** 32. Heterogeneity analysis of VAS.

| **t1** | **t2** | **I^2^.pair(%)** | **I^2^.cons(%)** | **Incons.p** |
| --- | --- | --- | --- | --- |
| CTRL | ZOL | 99.59 | 99.57 | NA |
| CTRL | TPTD | 99.62 | 97.63 | 0.78 |
| CTRL | DEN | NA | 84.65 | 0.73 |
| TPTD | DEN | NA | 92.97 | 0.73 |

VAS = Visual Analog Scale; CTRL = Background therapy control (Ca/VitD ± placebo); ZOL = Zoledronic acid; TPTD = Teriparatide; DEN = Denosumab. NA = Not Applicable.

**Supplementary Table** 33. Ranking probabilities for VAS.

| **Intervention** | **Rank 1** | **Rank 2** | **Rank 3** | **Rank 4** |
| --- | --- | --- | --- | --- |
| CTRL | 0.06 | 0.47 | 0.38 | 0.09 |
| ZOL | 0.70 | 0.18 | 0.07 | 0.04 |
| TPTD | 0.03 | 0.08 | 0.23 | 0.67 |
| DEN | 0.21 | 0.27 | 0.32 | 0.20 |

VAS = Visual Analog Scale; CTRL = Background therapy control (Ca/VitD ± placebo); ZOL = Zoledronic acid; TPTD = Teriparatide; DEN = Denosumab.

**Supplementary Table** 34. Leave-one-out sensitivity analysis of VAS.

| **Removed study** | **Key random-effects result** | **Rank-probability ranking** | **Interpretation** |
| --- | --- | --- | --- |
| Li Y et al. (2016) | TPTD vs CTRL significant: -0.96 (-1.51, -0.41); ZOL -0.04 (-0.74, 0.67). | TPTD > DEN > ZOL > CTRL | Heterogeneity high; ranking differed from the primary analysis. |
| Liu Z et al. (2019) | TPTD vs CTRL significant: -0.95 (-1.57, -0.32); ZOL -0.39 (-1.41, 0.63). | TPTD > ZOL > DEN > CTRL | Heterogeneity high; ranking sensitive. |
| Koutalos AA et al. (2022) | TPTD vs CTRL significant: -0.95 (-1.57, -0.32); ZOL -0.37 (-1.29, 0.56). | TPTD > ZOL > DEN > CTRL | Heterogeneity high. |
| Kim SJ et al. (2018) | TPTD vs CTRL significant: -1.30 (-1.81, -0.80); ZOL -0.17 (-0.70, 0.37). | TPTD > DEN > ZOL > CTRL | Heterogeneity decreased but remained notable. |
| Kim SJ et al. (2019) | TPTD vs CTRL significant: -0.86 (-1.58, -0.14); ZOL -0.23 (-0.96, 0.50). | TPTD > ZOL > DEN > CTRL | Heterogeneity high. |
| Hosseini Khameneh et al. (2023) | TPTD vs CTRL no longer significant: -0.86 (-1.77, 0.05); ZOL -0.27 (-1.15, 0.60). | TPTD > ZOL > DEN > CTRL | TPTD estimate sensitive to this study. |
| Ooi CS et al. (2024) | TPTD vs CTRL significant: -0.77 (-1.35, -0.20); DEN -0.61 (-1.58, 0.36). | TPTD > DEN > ZOL > CTRL | Heterogeneity decreased but remained notable. |
| Lu X et al. (2025) | TPTD vs CTRL significant: -0.77 (-1.35, -0.20); ZOL -0.20 (-0.83, 0.43). | TPTD > ZOL > CTRL > DEN | Heterogeneity decreased; DEN comparison unstable. |

VAS = Visual Analog Scale; MD = mean difference; CI = confidence interval; CTRL = Background therapy control (Ca/VitD ± placebo); TPTD = teriparatide; DEN = denosumab; ZOL = zoledronic acid. Lower VAS indicates less pain. Rankings were based on rank probabilities recalculated after each leave-one-out analysis. > indicates a more favorable rank-probability ranking, not necessarily a statistically significant difference.

**Supplementary Table** 35. GRADE certainty profile for 12-month hip refracture rate.

| **Comparison** | | | **Nature of the evidence** | **Confidence** | **Downgrading due to** |
| --- | --- | --- | --- | --- | --- |
| ZOL | VS | DEN | Indirect | Very low | Study limitation;Imprecision;Indirectness |
|  | VS | TPTD | Indirect | Very low | Study limitation;Imprecision;Indirectness |
|  | VS | ALN+TPTD | Indirect | Very low | Study limitation;Imprecision;Indirectness |
|  | VS | CTRL | Mixed | low | Study limitation;Imprecision |
| DEN | VS | TPTD | Indirect | Very low | Study limitation;Imprecision;Indirectness |
|  | VS | ALN+TPTD | Indirect | Very low | Study limitation;Imprecision;Indirectness |
|  | VS | CTRL | Mixed | low | Study limitation;Imprecision |
| TPTD | VS | ALN+TPTD | Indirect | Very low | Study limitation;Imprecision;Indirectness |
|  | VS | CTRL | Mixed | low | Study limitation;Imprecision |
| ALN+TPTD | VS | CTRL | Mixed | low | Study limitation;Imprecision |

**Supplementary Table** 36. GRADE certainty profile for 12-month overall refracture rate.

| **Comparison** | | | **Nature of the evidence** | **Confidence** | **Downgrading due to** |
| --- | --- | --- | --- | --- | --- |
| ALN | VS | ZOL | Indirect | Very low | Study limitation;Imprecision;Indirectness |
|  | VS | DEN | Indirect | Very low | Study limitation;Imprecision;Indirectness |
|  | VS | TPTD | Indirect | Very low | Study limitation;Imprecision;Indirectness |
|  | VS | ALN+TPTD | Indirect | Very low | Study limitation;Imprecision;Indirectness |
|  | VS | CTRL | Mixed | low | Study limitation;Imprecision |
| ZOL | VS | DEN | Indirect | Very low | Study limitation;Imprecision;Indirectness |
|  | VS | TPTD | Indirect | Very low | Study limitation;Imprecision;Indirectness |
|  | VS | ALN+TPTD | Indirect | Very low | Study limitation;Imprecision;Indirectness |
|  | VS | CTRL | Mixed | low | Study limitation;Imprecision |
| DEN | VS | TPTD | Indirect | Very low | Study limitation;Imprecision;Indirectness |
|  | VS | ALN+TPTD | Indirect | Very low | Study limitation;Imprecision;Indirectness |
|  | VS | CTRL | Mixed | low | Study limitation;Imprecision |
| TPTD | VS | ALN+TPTD | Indirect | Very low | Study limitation;Imprecision;Indirectness |
|  | VS | CTRL | Mixed | low | Study limitation;Imprecision |
| ALN+TPTD | VS | CTRL | Mixed | low | Study limitation;Imprecision |

**Supplementary Table** 37. GRADE certainty profile for 24-month overall refracture rate.

| **Comparison** | | | **Nature of the evidence** | **Confidence** | **Downgrading due to** |
| --- | --- | --- | --- | --- | --- |
| TPTD | VS | DEN | Indirect | Very low | Study limitation;Imprecision;Indirectness |
|  | VS | ZOL | Indirect | Very low | Study limitation;Imprecision;Indirectness |
|  | VS | CTRL | Mixed | low | Study limitation; Imprecision |
| DEN | VS | ZOL | Indirect | Very low | Study limitation;Imprecision;Indirectness |
|  | VS | CTRL | Mixed | low | Study limitation; Imprecision |
| ZOL | VS | CTRL | Mixed | low | Study limitation: Imprecision |

**Supplementary Table** 38. GRADE certainty profile for hip BMD (g/cm²).

| **Comparison** | | | **Nature of the evidence** | **Confidence** | **Downgrading due to** |
| --- | --- | --- | --- | --- | --- |
| ZOL | VS | TPTD | Indirect | Very low | Study limitation;Imprecision;Indirectness |
|  | VS | RIS | Indirect | Very low | Study limitation;Imprecision;Indirectness |
|  | VS | CTRL | Mixed | low | Study limitation; Imprecision |
| TPTD | VS | RIS | Indirect | Very low | Study limitation;Imprecision;Indirectness |
|  | VS | CTRL | Mixed | low | Study limitation;Imprecision |
| RIS | VS | CTRL | Mixed | low | Study limitation;Imprecision |

**Supplementary Table** 39. GRADE certainty profile for hip BMD (T-score).

| **Comparison** | | | **Nature of the evidence** | **Confidence** | **Downgrading due to** |
| --- | --- | --- | --- | --- | --- |
| ALN | VS | TPTD | Indirect | Very low | Study limitation;Imprecision;Indirectness |
|  | VS | DEN | Indirect | Very low | Study limitation;Imprecision;Indirectness |
|  | VS | CTRL | Mixed | low | Study limitation;Imprecision |
| TPTD | VS | DEN | Indirect | Very low | Study limitation;Imprecision;Indirectness |
|  | VS | CTRL | Mixed | low | Study limitation;Imprecision |
| DEN | VS | CTRL | Mixed | low | Study limitation;Imprecision |

**Supplementary Table** 40. GRADE certainty profile for HHS.

| **Comparison** | | | **Nature of the evidence** | **Confidence** | **Downgrading due to** |
| --- | --- | --- | --- | --- | --- |
| ALN | VS | DEN | Indirect | Very low | Study limitation;Inconsistency |
|  | VS | TPTD | Indirect | Very low | Study limitation;Inconsistency |
|  | VS | ZOL | Indirect | Very low | Study limitation;Inconsistency |
|  | VS | CTRL | Mixed | Very low | Study limitation;Inconsistency |
| DEN | VS | TPTD | Indirect | Very low | Study limitation;Inconsistency |
|  | VS | ZOL | Indirect | Very low | Study limitation;Inconsistency |
|  | VS | CTRL | Mixed | Very low | Study limitation;Inconsistency |
| TPTD | VS | ZOL | Indirect | Very low | Study limitation;Inconsistency |
|  | VS | CTRL | Mixed | Very low | Study limitation;Inconsistency |
| ZOL | VS | CTRL | Mixed | Very low | Study limitation;Inconsistency |

**Supplementary Table** 41. GRADE certainty profile for VAS.

| **Comparison** | | | **Nature of the evidence** | **Confidence** | **Downgrading due to** |
| --- | --- | --- | --- | --- | --- |
| ZOL | VS | DEN | Indirect | Very low | Study limitation;Inconsistency |
|  | VS | TPTD | Indirect | Very low | Study limitation;Inconsistency |
|  | VS | CTRL | Mixed | Very low | Study limitation;Inconsistency |
| DEN | VS | TPTD | Indirect | Very low | Study limitation;Inconsistency |
|  | VS | CTRL | Mixed | Very low | Study limitation;Inconsistency |
| TPTD | VS | CTRL | Mixed | Very low | Study limitation;Inconsistency |
