# Supplementary material for: Effects of different anti-osteoporosis medications on re-fracture risk, bone mineral density, and functional recovery following hip fragility fracture surgery: a systematic review and network meta-analysis
Source: Front Endocrinol (Lausanne). 2026 Oct 20;17:1949781. doi: 10.3389/fendo.2026.1949781 (PMC13727487; doi:10.3389/fendo.2026.1949781)
Supplement: Supplementary file 2 [file Supplementaryfile2.docx]

***Supplementary Material 2***

Supplementary Table 42. Raw data for this study (VAS)

|  | **Study and year** | **Treatments** | | | | **VAS** | | | | | | |  | **Follow-up time** |
| --- | --- | --- | --- | --- | --- | --- | --- | --- | --- | --- | --- | --- | --- | --- |
|  |  | **Group 1** |  | **Group 2** |  | **Group 1** | | | **Group 2** | | | |  |  |
|  |  |  |  |  |  | **Preoperative** |  | **Postoperation** |  | **Preoperative** |  | **Postoperation** |  |  |
| 1 | Kim SJ, et al. (2018) | TPTD |  | CTRL |  |  |  | 2.45±1.625 |  |  |  | 2.28±1.75 |  | 6 months |
| 2 | Koutalos AA, et al. (2022) | ZOL |  | CTRL |  |  |  | 1.3±1.6 |  |  |  | 1.3±1.3 |  | 12 months |
| 3 | Li Y, et al. (2016) | ZOL |  | CTRL |  | 8.2±3.5 |  | 3±2.8 |  | 7.8±3.8 |  | 4.3±3.2 |  | 12 months |
| 4 | Hosseini Khameneh M, et al. (2023) | TPTD |  | CTRL |  |  |  | 2.8±0.05 |  |  |  | 3.88±0.08 |  | 12 months |
| 5 | Ooi CS, et al. (2024) | TPTD |  | DEN |  | 7.3±0.9 |  | 2.8±0.7 |  | 7.2±0.9 |  | 4.2±0.9 |  | 24 months |
| 6 | Kim SJ, et al. (2019) | TPTD |  | CTRL |  |  |  | 2.1±1.625 |  |  |  | 3.3±1.75 |  | 6 months |
| 7 | Liu Z, et al. (2019) | ZOL |  | CTRL |  | 7.12±0.67 |  | 1.22±0.67 |  | 7.18±0.36 |  | 1.28±0.35 |  | 24 months |
| 8 | Lu X, et al. (2025) | DEN |  | CTRL |  | 6±0.96 |  | 1.68±0.93 |  | 5.95±1.04 |  | 2.29±0.97 |  | 12 months |

CTRL = Background therapy control (Ca/VitD ± placebo); TPTD = Teriparatide; DEN = Denosumab; ALN = Alendronate; ZOL = Zoledronic acid.

Supplementary Table 43. Raw data for this study (HHS)

|  | **Study and year** | **Treatments** | | | | **HHS** | | | | | | |  | **Follow-up time** |
| --- | --- | --- | --- | --- | --- | --- | --- | --- | --- | --- | --- | --- | --- | --- |
|  |  | **Group 1** |  | **Group 2** |  | **Group 1** | | | **Group 2** | | | |  |  |
|  |  |  |  |  |  | **Preoperative** |  | **Postoperation** |  | **Preoperative** |  | **Postoperation** |  |  |
| 1 | Tanavalee C, et al. (2025) | TPTD |  | CTRL |  | 78.21±15.51 |  | 74.9±17.8 |  | 75.4±16.68 |  | 75±16.7 |  | 6 months |
| 2 | Wang KM, et al. (2022) | ALN |  | CTRL |  | 34.18±3.21 |  | 91.23±4.52 |  | 34.25±3.05 |  | 83.36±3.64 |  | 12 months |
| 3 | Kim SJ, et al. (2018) | TPTD |  | CTRL |  |  |  | 65.4±16.5 |  |  |  | 62.2±15 |  | 6 months |
| 4 | Li Y, et al. (2016) | ZOL |  | CTRL |  |  |  | 81.12±7.9 |  |  |  | 81.62±5.4 |  | 12 months |
| 5 | Huang TW, et al. (2016) | TPTD |  | CTRL |  | 15.4±8.4 |  | 81.5±9.9 |  | 15.9±9.1 |  | 83.6±7.5 |  | 12months |
| 6 | Hosseini Khameneh M, et al. (2023) | TPTD |  | CTRL |  | 64.02±6 |  | 85.12±3 |  | 65.07±3 |  | 75.35±4 |  | 12 months |
| 7 | Kim SJ, et al. (2019) | TPTD |  | CTRL |  |  |  | 70.4±16.5 |  |  |  | 60.4±16.5 |  | 6 months |
| 8 | Lu X, et al. (2025) | DEN |  | CTRL |  | 43.05±6.19 |  | 86.24±3.58 |  | 42.95±6.26 |  | 82.87±4.11 |  | 12 months |

CTRL = Background therapy control (Ca/VitD ± placebo); TPTD = Teriparatide; DEN = Denosumab; ALN = Alendronate; ZOL = Zoledronic acid.

Supplementary Table 44. Raw data for this study (BMD, g/cm²)

|  | **Study and year** | **Treatments** | | | | **BMD (g/cm²)** | | | | | | |  | **Follow-up time** |
| --- | --- | --- | --- | --- | --- | --- | --- | --- | --- | --- | --- | --- | --- | --- |
|  |  | **Group 1** |  | **Group 2** |  | **Group 1** | | | **Group 2** | | | |  |  |
|  |  |  |  |  |  | **Preoperative** |  | **Postoperation** |  | **Preoperative** |  | **Postoperation** |  |  |
| 1 | Li Y, et al. (2016) | ZOL |  | CTRL |  | 0.65±0.13 |  | 0.76±0.14 |  | 0.69±0.11 |  | 0.7±0.1 |  | 12 months |
| 2 | Aggarwal HO, et al. (2025) | TPTD |  | CTRL |  | 0.671±0.087 |  | 0.705±0.066 |  | 0.651±0.087 |  | 0.656±0.087 |  | 6 months |
| 3 | Rana A, et al. (2021) | TPTD |  | CTRL |  | 0.565±0.082 |  | 0.575±0.084 |  | 0.549±0.077 |  | 0.554±0.078 |  | 6 months |
| 4 | Malouf-Sierra J, et al. (2017) | TPTD |  | RIS |  | 0.67±0.098 |  | 0.687±0.101 |  | 0.672±0.113 |  | 0.675±0.114 |  | 12 months |

CTRL = Background therapy control (Ca/VitD ± placebo); TPTD = Teriparatide; ZOL = Zoledronic acid; RIS = Risedronate.

Supplementary Table 45. Raw data for this study (BMD, T-score)

|  | **Study and year** | **Treatments** | | | | **BMD (T-score)** | | | | | | |  | **Follow-up time** |
| --- | --- | --- | --- | --- | --- | --- | --- | --- | --- | --- | --- | --- | --- | --- |
|  |  | **Group 1** |  | **Group 2** |  | **Group 1** | | | **Group 2** | | | |  |  |
|  |  |  |  |  |  | **Preoperative** |  | **Postoperation** |  | **Preoperative** |  | **Postoperation** |  |  |
| 1 | Wang KM, et al. (2022) | ALN |  | CTRL |  | -2.60±0.15 |  | -1.37±0.10 |  | -2.61±0.18 |  | -2.08±0.13 |  | 12 months |
| 2 | Ooi CS, et al. (2024) | TPTD |  | DEN |  | -3.00±0.60 |  | -2.30±0.60 |  | -3.10±0.90 |  | -2.60±0.70 |  | 24 months |
| 3 | Lu X, et al. (2025) | DEN |  | CTRL |  | -2.91±0.34 |  | -1.49±0.61 |  | -2.87±0.37 |  | -1.98±0.52 |  | 12 months |
| 4 | Aggarwal HO, et al. (2025) | TPTD |  | CTRL |  | -3.04±0.43 |  | -2.83±0.43 |  | -3.17±0.73 |  | -3.15±0.73 |  | 6 months |

CTRL = Background therapy control (Ca/VitD ± placebo); TPTD = Teriparatide; DEN = Denosumab; ALN = Alendronate.

Supplementary Table 46. Raw data for this study (Refracture rate 12 months)

|  | **Study and year** | **Treatments** | | | | |  | **Refracture rate, n (%)** | | | | |  | **Follow-up time** |
| --- | --- | --- | --- | --- | --- | --- | --- | --- | --- | --- | --- | --- | --- | --- |
|  |  | **Group 1** |  | **Group 2** |  | **Group 3** |  | **Group 1** |  | **Group 2** |  | **Group 3** |  |  |
| 1 | Li Y, et al. (2016) | ZOL |  | CTRL |  |  |  | 0(0.00) |  | 4(13.33) |  |  |  | 12 months |
| 2 | Huang TW, et al. (2016) | TPTD |  | CTRL |  |  |  | 5(12.50) |  | 7(13.46) |  |  |  | 12 months |
| 3 | Lu X, et al. (2025) | DEN |  | CTRL |  |  |  | 1(2.63) |  | 8(21.05) |  |  |  | 12 months |
| 4 | Wang Y, et al. (2025) | DEN |  | CTRL |  |  |  | 1(1.10) |  | 10(10.99) |  |  |  | 12 months |
| 5 | Wang KM, et al. (2022) | ALN |  | CTRL |  |  |  | 0(0.00) |  | 4(6.35) |  |  |  | 12 months |
| 6 | Huang TW, et al. (2016) | TPTD |  | CTRL |  | ALN+TPTD |  | 10(21.28) |  | 27(32.53) |  | 10(16.59) |  | 12 months |

CTRL = Background therapy control (Ca/VitD ± placebo); TPTD = Teriparatide; DEN = Denosumab; ALN = Alendronate; ZOL = Zoledronic acid; ALN+TPTD = pre-fracture alendronate exposure followed by postoperative teriparatide treatment.

Supplementary Table 47. Raw data for this study (Hip refracture rate 12 months)

|  | **Study and year** | **Treatments** | | | | |  | **Refracture rate, n (%)** | | | | |  | **Follow-up time** |
| --- | --- | --- | --- | --- | --- | --- | --- | --- | --- | --- | --- | --- | --- | --- |
|  |  | **Group 1** |  | **Group 2** |  | **Group 3** |  | **Group 1** |  | **Group 2** |  | **Group 3** |  |  |
| 1 | Li Y, et al. (2016) | ZOL |  | CTRL |  |  |  | 0(0.00) |  | 2(6.67) |  |  |  | 12 months |
| 2 | Huang TW, et al. (2016) | TPTD |  | CTRL |  |  |  | 1(2.50) |  | 1(1.92) |  |  |  | 12 months |
| 3 | Lu X, et al. (2025) | DEN |  | CTRL |  |  |  | 1(2.63) |  | 5(13.16) |  |  |  | 12 months |
| 4 | Wang Y, et al. (2025) | DEN |  | CTRL |  |  |  | 1(1.10) |  | 10(10.99) |  |  |  | 12 months |
| 5 | Huang TW, et al. (2016) | TPTD |  | CTRL |  | ALN+TPTD |  | 2(4.26) |  | 6(7.23) |  | 1(1.69) |  | 12 months |

CTRL = Background therapy control (Ca/VitD ± placebo); TPTD = Teriparatide; DEN = Denosumab; ALN = Alendronate; ZOL = Zoledronic acid; ALN+TPTD = pre-fracture alendronate exposure followed by postoperative teriparatide treatment.

Supplementary Table 48. Raw data for this study (Refracture rate 24 months)

|  | **Study and year** | **Treatments** | | | | **Refracture rate, n (%)** | | | | **Follow-up time** |
| --- | --- | --- | --- | --- | --- | --- | --- | --- | --- | --- |
|  |  | **Group 1** |  | **Group 2** |  | **Group 1** |  | **Group 2** |  |  |
| 1 | Liu Z, et al. (2019) | ZOL |  | CTRL |  | 21(5.95) |  | 11(8.53) |  | 24 months |
| 2 | Kim SJ, et al. (2022) | DEN |  | ZOL |  | 5(3.09) |  | 3(2.50) |  | 24 months |
| 3 | Ooi CS, et al. (2024) | TPTD |  | DEN |  | 2(2.94) |  | 6(10.34) |  | 24 months |
| 4 | Wang Y, et al. (2025) | DEN |  | CTRL |  | 2(2.20) |  | 14(15.38) |  | 24 months |

CTRL = Background therapy control (Ca/VitD ± placebo); TPTD = Teriparatide; DEN = Denosumab; ZOL = Zoledronic acid.

Supplementary Table 49. Meta-analysis data of the study and year (HHS)

| **Study and year** | **Treatment** | **mean** | **std.dev** | **Samplesize** | **Follow-up time** |
| --- | --- | --- | --- | --- | --- |
| Kim SJ, et al. (2018) | CTRL | 62.2 | 15 | 50 | 6 months |
| Kim SJ, et al. (2018) | TPTD | 65.4 | 16.5 | 46 | 6 months |
| Kim SJ, et al. (2019) | CTRL | 60.4 | 16.5 | 60 | 6 months |
| Kim SJ, et al. (2019) | TPTD | 70.4 | 16.5 | 52 | 6 months |
| Hosseini Khameneh M, et al. (2023) | CTRL | 75.35 | 4 | 32 | 12 months |
| Hosseini Khameneh M, et al. (2023) | TPTD | 85.12 | 3 | 40 | 12 months |
| Tanavalee C, et al. (2025) | CTRL | 75 | 16.7 | 25 | 6 months |
| Tanavalee C, et al. (2025) | TPTD | 74.9 | 17.8 | 25 | 6 months |
| Lu X, et al. (2025) | CTRL | 82.87 | 4.11 | 38 | 12 months |
| Lu X, et al. (2025) | DEN | 86.24 | 3.58 | 38 | 12 months |
| Wang KM, et al. (2022) | CTRL | 83.36 | 3.64 | 63 | 12months |
| Wang KM, et al. (2022) | ALN | 91.23 | 4.52 | 63 | 12months |
| Huang TW, et al. (2016) | CTRL | 83.6 | 7.5 | 52 | 12months |
| Huang TW, et al. (2016) | TPTD | 81.5 | 9.9 | 40 | 12months |
| Li Y, et al. (2016) | CTRL | 81.62 | 5.4 | 30 | 12 months |
| Li Y, et al. (2016) | ZOL | 81.12 | 7.9 | 30 | 12 months |

HHS = Harris Hip Score; CTRL = Background therapy control (Ca/VitD ± placebo); TPTD = Teriparatide; DEN = Denosumab; ALN = Alendronate; ZOL = Zoledronic acid.

Supplementary Table 50. Meta-analysis data of the study and year (VAS)

| **Study and year** | **Treatment** | **mean** | **std.dev** | **Samplesize** | **Follow-up time** |
| --- | --- | --- | --- | --- | --- |
| Li Y, et al. (2016) | CTRL | 4.3 | 3.2 | 30 | 12 months |
| Li Y, et al. (2016) | ZOL | 3 | 2.8 | 30 | 12 months |
| Liu Z, et al. (2019) | CTRL | 1.28 | 0.35 | 109 | 24 months |
| Liu Z, et al. (2019) | ZOL | 1.22 | 0.67 | 332 | 24 months |
| Koutalos AA, et al. (2022) | CTRL | 1.3 | 1.3 | 46 | 12 months |
| Koutalos AA, et al. (2022) | ZOL | 1.3 | 1.6 | 45 | 12 months |
| Kim SJ, et al. (2018) | CTRL | 2.28 | 1.75 | 50 | 6 months |
| Kim SJ, et al. (2018) | TPTD | 2.45 | 1.625 | 46 | 6 months |
| Kim SJ, et al. (2019) | CTRL | 3.3 | 1.75 | 60 | 6 months |
| Kim SJ, et al. (2019) | TPTD | 2.1 | 1.625 | 52 | 6 months |
| Hosseini Khameneh M, et al. (2023) | CTRL | 3.88 | 0.08 | 32 | 12 months |
| Hosseini Khameneh M, et al. (2023) | TPTD | 2.8 | 0.05 | 40 | 12 months |
| Ooi CS, et al. (2024) | DEN | 4.2 | 0.9 | 58 | 24 months |
| Ooi CS, et al. (2024) | TPTD | 2.8 | 0.7 | 68 | 24 months |
| Lu X, et al. (2025) | CTRL | 2.29 | 0.97 | 38 | 12 months |
| Lu X, et al. (2025) | DEN | 1.68 | 0.93 | 38 | 12 months |

VAS = Visual Analog Scale; CTRL = Background therapy control (Ca/VitD ± placebo); ZOL = Zoledronic acid; TPTD = Teriparatide; DEN = Denosumab.

Supplementary Table 51. Meta-analysis data of the study and year (Refracture rate 12 months)

| **Study and year** | **Treatment** | **d** | **n** | **Follow-up time** |
| --- | --- | --- | --- | --- |
| Li Y, et al. (2016) | ZOL | 0 | 30 | 12 months |
| Li Y, et al. (2016) | CTRL | 4 | 30 | 12 months |
| Huang TW, et al. (2016) | TPTD | 5 | 40 | 12 months |
| Huang TW, et al. (2016) | CTRL | 7 | 52 | 12 months |
| Lu X, et al. (2025) | DEN | 1 | 38 | 12 months |
| Lu X, et al. (2025) | CTRL | 8 | 38 | 12 months |
| Wang Y, et al. (2025) | DEN | 1 | 91 | 12 months |
| Wang Y, et al. (2025) | CTRL | 10 | 91 | 12 months |
| Wang KM, et al. (2022) | ALN | 0 | 63 | 12 months |
| Wang KM, et al. (2022) | CTRL | 4 | 63 | 12 months |
| Huang TW, et al. (2016) | TPTD | 10 | 47 | 12 months |
| Huang TW, et al. (2016) | CTRL | 27 | 83 | 12 months |
| Huang TW, et al. (2016) | ALN+TPTD | 10 | 59 | 12 months |

CTRL = Background therapy control (Ca/VitD ± placebo); ZOL = Zoledronic acid; DEN = Denosumab; TPTD = Teriparatide; ALN+TPTD = pre-fracture alendronate exposure followed by postoperative teriparatide treatment; ALN = Alendronate.

Supplementary Table 52. Meta-analysis data of the study and year (BMD (g/cm²))

| **Study and year** | **Treatment** | **mean** | **std.dev** | **Samplesize** | **Follow-up time** |
| --- | --- | --- | --- | --- | --- |
| Li Y, et al. (2016) | ZOL | 0.11 | 0.135 | 30 | 12 months |
| Li Y, et al. (2016) | CTRL | 0.01 | 0.105 | 30 | 12 months |
| Rana A, et al. (2021) | TPTD | 0.01 | 0.083 | 15 | 6 months |
| Rana A, et al. (2021) | CTRL | 0.005 | 0.078 | 15 | 6 months |
| Malouf-Sierra J, et al. (2017) | TPTD | 0.017 | 0.1 | 86 | 12 months |
| Malouf-Sierra J, et al. (2017) | RIS | 0.003 | 0.114 | 85 | 12 months |
| Aggarwal HO, et al. (2025) | TPTD | 0.034 | 0.074 | 20 | 6 months |
| Aggarwal HO, et al. (2025) | CTRL | 0.005 | 0.087 | 20 | 6 months |

BMD (g/cm²)= Bone Mineral Density (grams per square centimeter); CTRL = Background therapy control (Ca/VitD ± placebo); ZOL = Zoledronic acid; TPTD = Teriparatide; RIS = Risedronate.

Supplementary Table 53. Meta-analysis data of the study and year (BMD (T-score))

| **Study and year** | **Treatment** | **mean** | **std.dev** | **Samplesize** | **Follow-up time** |
| --- | --- | --- | --- | --- | --- |
| Ooi CS, et al. (2024) | TPTD | 0.7 | 0.465 | 68 | 24 months |
| Ooi CS, et al. (2024) | DEN | 0.5 | 0.646 | 58 | 24 months |
| Lu X, et al. (2025) | DEN | 1.42 | 0.445 | 38 | 12 months |
| Lu X, et al. (2025) | CTRL | 0.89 | 0.371 | 38 | 12 months |
| Wang KM, et al. (2022) | ALN | 1.23 | 0.107 | 63 | 12 months |
| Wang KM, et al. (2022) | CTRL | 0.53 | 0.128 | 63 | 12 months |
| Aggarwal HO, et al. (2025) | TPTD | 0.21 | 0.333 | 20 | 6 months |
| Aggarwal HO, et al. (2025) | CTRL | 0.02 | 0.566 | 20 | 6 months |

BMD (T-score)= Bone Mineral Density T-score; CTRL = Background therapy control (Ca/VitD ± placebo); TPTD = Teriparatide; DEN = Denosumab; ALN = Alendronate.

Supplementary Table 54. Meta-analysis data of the study and year (Hip refracture rate 12 months)

| **Study and year** | **Treatment** | **d** | **n** | **Follow-up time** |
| --- | --- | --- | --- | --- |
| Li Y, et al. (2016) | CTRL | 2 | 30 | 12 months |
| Li Y, et al. (2016) | ZOL | 0 | 30 | 12 months |
| Huang TW, et al. (2016) | CTRL | 1 | 52 | 12 months |
| Huang TW, et al. (2016) | TPTD | 1 | 40 | 12 months |
| Lu X, et al. (2025) | CTRL | 5 | 38 | 12 months |
| Lu X, et al. (2025) | DEN | 1 | 38 | 12 months |
| Wang Y, et al. (2025) | CTRL | 10 | 91 | 12 months |
| Wang Y, et al. (2025) | DEN | 1 | 91 | 12 months |
| Huang TW, et al. (2016) | CTRL | 6 | 83 | 12 months |
| Huang TW, et al. (2016) | TPTD | 2 | 47 | 12 months |
| Huang TW, et al. (2016) | ALN+TPTD | 1 | 59 | 12 months |

CTRL = Background therapy control (Ca/VitD ± placebo); ZOL = Zoledronic acid; DEN = Denosumab; TPTD = Teriparatide; ALN = Alendronate. ALN+TPTD = pre-fracture alendronate exposure followed by postoperative teriparatide treatment.

Supplementary Table 55. Meta-analysis data of the study and year (Refracture rate 24 months)

| **Study and year** | **Treatment** | **d** | **n** | **Follow-up time** |
| --- | --- | --- | --- | --- |
| Liu Z, et al. (2019) | CTRL | 11 | 129 | 24 months |
| Liu Z, et al. (2019) | ZOL | 21 | 353 | 24 months |
| Kim SJ, et al. (2022) | DEN | 5 | 162 | 24 months |
| Kim SJ, et al. (2022) | ZOL | 3 | 120 | 24 months |
| Ooi CS, et al. (2024) | TPTD | 2 | 68 | 24 months |
| Ooi CS, et al. (2024) | DEN | 6 | 58 | 24 months |
| Wang Y, et al. (2025) | CTRL | 14 | 91 | 24 months |
| Wang Y, et al. (2025) | DEN | 2 | 91 | 24 months |

CTRL = Background therapy control (Ca/VitD ± placebo); TPTD = Teriparatide; DEN = Denosumab; ZOL = Zoledronic acid.
