# Supplementary material for: Effects of different anti-osteoporosis medications on re-fracture risk, bone mineral density, and functional recovery following hip fragility fracture surgery: a systematic review and network meta-analysis
Source: Front Endocrinol (Lausanne). 2026 Oct 20;17:1949781. doi: 10.3389/fendo.2026.1949781 (PMC13727487; doi:10.3389/fendo.2026.1949781)
Supplement: Supplementary file 3 [file Supplementaryfile3.docx]

**Supplementary Material 3**

**MCMC Trace and Posterior Density Diagnostics**

Outcome-specific Bayesian network meta-analysis models

**Supplementary figures**

Figure S1: 12-month overall refracture

Figure S2: 12-month hip refracture

Figure S3: 24-month overall refracture

Figure S4: HHS

Figure S5: VAS

Figure S6: BMD (g/cm²)

Figure S7: BMD (T-score)

**Figure note**

Note: In each figure, trace plots show the sampled values across four MCMC chains, whereas density plots show the corresponding posterior distributions. Labels beginning with "d." denote outcome-specific treatment-effect parameters, while "sd.d" denotes the between-study heterogeneity standard deviation. Different colors represent the four MCMC chains. "N" indicates the number of retained posterior samples, and "bandwidth" indicates the kernel density bandwidth. Numerical convergence statistics, including PSRF and ESS, are reported in Supplementary Table S4.

**Supplementary Figure S1**

**12-month overall refracture**

MCMC trace and posterior density diagnostics

Primary Bayesian random-effects consistency model

Note: Broad or asymmetric posterior distributions in these 12-month refracture networks reflect sparse comparisons and zero-event data. These diagnostics should be interpreted together with the PSRF and ESS values reported in Supplementary Table S4.

**Supplementary Figure S2**

**12-month hip refracture**

MCMC trace and posterior density diagnostics

Primary Bayesian random-effects consistency model

Note: Broad or asymmetric posterior distributions in these 12-month refracture networks reflect sparse comparisons and zero-event data. These diagnostics should be interpreted together with the PSRF and ESS values reported in Supplementary Table S4.

**Supplementary Figure S3**

**24-month overall refracture**

MCMC trace and posterior density diagnostics

Primary Bayesian random-effects consistency model

**Supplementary Figure S4**

**HHS**

MCMC trace and posterior density diagnostics

Primary Bayesian random-effects consistency model

**Supplementary Figure S5**

**VAS**

MCMC trace and posterior density diagnostics

Primary Bayesian random-effects consistency model

**Supplementary Figure S6**

**BMD (g/cm²)**

MCMC trace and posterior density diagnostics

Primary Bayesian random-effects consistency model

**Supplementary Figure S7**

**BMD (T-score)**

MCMC trace and posterior density diagnostics

Primary Bayesian random-effects consistency model
